# Establishment and Validation of Computational Model for MT1-MMP Dependent ECM Degradation and Intervention Strategies

**DOI:** 10.1371/journal.pcbi.1002479

**Published:** 2012-04-12

**Authors:** Daisuke Hoshino, Naohiko Koshikawa, Takashi Suzuki, Vito Quaranta, Alissa M. Weaver, Motoharu Seiki, Kazuhisa Ichikawa

**Affiliations:** 1 Division of Cancer Cell Research, Institute of Medical Science, University of Tokyo, Minato-ku, Tokyo, Japan; 2 Division of Mathematical Science, Graduate School of Engineering Science, Osaka University, Toyonaka, Osaka, Japan; 3 JST, CREST, Chiyoda-ku, Tokyo, Japan; 4 Department of Cancer Biology, Vanderbilt University Medical Center, Nashville, Tennessee, United States of America; 5 Division of Mathematical Oncology, Institute of Medical Science, University of Tokyo, Minato-ku, Tokyo, Japan; Johns Hopkins University, United States of America

## Abstract

MT1-MMP is a potent invasion-promoting membrane protease employed by aggressive cancer cells. MT1-MMP localizes preferentially at membrane protrusions called invadopodia where it plays a central role in degradation of the surrounding extracellular matrix (ECM). Previous reports suggested a role for a continuous supply of MT1-MMP in ECM degradation. However, the turnover rate of MT1-MMP and the extent to which the turnover contributes to the ECM degradation at invadopodia have not been clarified. To approach this problem, we first performed FRAP (Fluorescence Recovery after Photobleaching) experiments with fluorescence-tagged MT1-MMP focusing on a single invadopodium and found very rapid recovery in FRAP signals, approximated by double-exponential plots with time constants of 26 s and 259 s. The recovery depended primarily on vesicle transport, but negligibly on lateral diffusion. Next we constructed a computational model employing the observed kinetics of the FRAP experiments. The simulations successfully reproduced our FRAP experiments. Next we inhibited the vesicle transport both experimentally, and in simulation. Addition of drugs inhibiting vesicle transport blocked ECM degradation experimentally, and the simulation showed no appreciable ECM degradation under conditions inhibiting vesicle transport. In addition, the degree of the reduction in ECM degradation depended on the degree of the reduction in the MT1-MMP turnover. Thus, our experiments and simulations have established the role of the rapid turnover of MT1-MMP in ECM degradation at invadopodia. Furthermore, our simulations suggested synergetic contributions of proteolytic activity and the MT1-MMP turnover to ECM degradation because there was a nonlinear and marked reduction in ECM degradation if both factors were reduced simultaneously. Thus our computational model provides a new in silico tool to design and evaluate intervention strategies in cancer cell invasion.

## Author Summary

Prevention of invasion is important in cancer therapy. MT1-MMP is a membrane protein involved in degradation of ECM (extracellular matrix) that is highly expressed at invadopodia, which are small protrusions of cancer cells. ECM degradation by MT1-MMP at invadopodia is hypothesized as the initial step of cancer cell invasion. However, MT1-MMP is inhibited by the endogenous inhibitor TIMP-2, so continuous turnover of MT1-MMP at the surface of invadopodia would be required. In agreement, it has been reported that the blockade of vesicle transport, which is one mechanism involved in the turnover, blocked the ECM degradation. However, the turnover rate of MT1-MMP at invadopodia and the extent to which the turnover is critical for the degradation of ECM have not been clarified. In this report we measured the turnover rate of MT1-MMP at a single invadopodium and found rapid turnover rates with time constants of 26 s and 259 s, which primarily depended on the vesicle transport. A computational model was constructed based on the observed kinetics. If we blocked the rapid turnover, the ECM degradation was blocked both experimentally and in simulations. These results established the role of the rapid turnover of MT1-MMP in the ECM degradation at invadopodia.

## Introduction

Some matrix metalloproteinases (MMPs) are proinvasive and employed by motile and invasive cells to degrade extracellular matrix (ECM). Among the 23 MMPs in mammals, integral membrane type MMPs, especially MT1-MMP, are believed to provide major contributions to cancer cell invasion. In fact, specific inhibition of MT1-MMP activity or knockdown of its expression suppresses not only cancer cell invasion in vitro but also tumor growth in mice [1], [2], [3], [4]. Therefore, MT1-MMP must be an important component of the cellular invasion machinery, and indeed, active ECM degradation is produced at structures called invadopodia [5], [6], which are specialized membrane protrusions extending into the ECM. Thus, invadopodia are hypothesized as machinery to degrade ECM at the initial stage of cancer cell invasion.

MT1-MMP was reported to be enriched in invadopodia [7], [8], [9]. In addition, the accumulation of MT1-MMP in invadopodia was followed by the degradation of ECM at the same sites [8]. These observations indicate that MT1-MMP plays a crucial role in ECM degradation at invadopodia [10]. However, the ECM-degrading activity of MT1-MMP will not be persistent, because cell-surface MT1-MMP is inactivated by TIMP-2 which is an endogenous MT1-MMP inhibitor that inhibits the proteolytic degradation of ECM. Therefore, a continuous supply of active MT1-MMP to the surface seems to be critical for maintaining ECM degrading [11].

Several reports have been published that support the importance of a continuous supply of MT1-MMP. It is reported that MT1-MMP is delivered to the invasion front of cells by vesicle transport in a 3D collagen matrix [12]. MT1-MMP is delivered to invadopodia by the exocyst complex, and the process is regulated by cortactin, IQGAP1 and RhoA [8], [13]. VAMP7, a member of the v-snare protein family, also regulates MT1-MMP transport to invadopodia, and MT1-MMP appears to be stored in late endosomal/lysosomal vesicles together with VAMP7 [14].

In addition to the supply of MT1-MMP to the surface, the internalization of MT1-MMP was also reported [15], [16]. MT1-MMP was colocated with clathrin, and the internalization proceeded through a clathrin-dependent pathway [16], [17], [18] or in a caveolae-mediated manner[19], [20]. Inhibition of caveolin-1 by siRNA was shown to block the MT1-MMP-dependent ECM degradation [21].

All these previous reports strongly suggest a crucial role of a continuous supply and internalization of surface MT1-MMP (i.e. the turnover of MT1-MMP), particularly by vesicle-mediated mechanisms. However, the turnover rate of MT1-MMP and the extent to which the turnover contributes to ECM degradation at invadopodia have not been clarified. In the present study, we attempted to measure experimentally the turnover rate of MT1-MMP at a single invadopodium, and to elucidate the role of the turnover in ECM degradation by computer simulations.

First, we analyzed the turnover rate of MT1-MMP at a single invadopodium by FRAP (Fluorescence Recovery after Photobleaching) experiments using a chimeric MT1-MMP with a pH-sensitive GFP protein (pHLuorin). The recovery of fluorescence was plotted in a double-exponential plot, which showed time constants of 26.0 s and 259 s. Based on these experiments, we constructed a computational model to analyze the role of the rapid MT1-MMP turnover. Simulations showed no appreciable ECM degradation when the rapid turnover was inhibited in our simulation experiments. This was also true in the actual experiments. In addition, simulations have shown a decrease in the ECM degradation concomitantly with an increased reduction of the turnover rate of MT1-MMP. Thus, we have quantified the rate of turnover of MT1-MMP at single invadopodia and shown its critical role in ECM degradation both experimentally and by computer simulations. Furthermore, our simulations have shown a synergetic effect on the inhibition of ECM degradation by simultaneous reductions of the MT1-MMP turnover rate and the vesicular content of it. Our computational model provides a new in silico tool to design and evaluate intervention strategies in cancer cell invasion.

## Results

### Vesicular transport is required for ECM degradation at invadopodia

**Figure 1:**
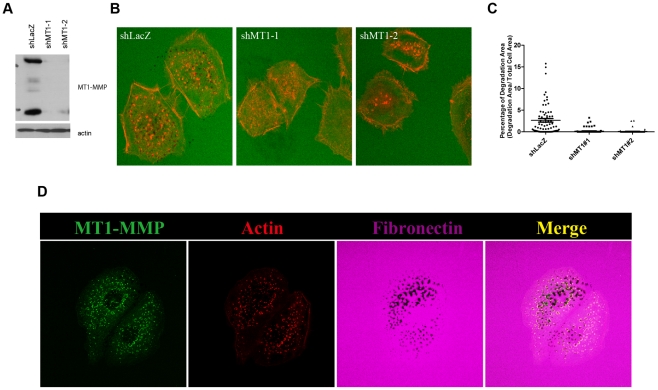
MT1-MMP is required for invadopodia-related ECM degradation. (A) Protein levels of MT1-MMP were assessed by Western blot analysis using anti-MT1-MMP antibody. Actin as a loading control. (B) Representative images show Dylight 633-labeled fibronectin (Green) and actin (Red). (C) Quantification of fibronectin degradation area in Figure 1 B (n = 3). (D) Representative images of MT1-Luo localization (Green), actin staining (Red) and Dylight 633-labeled fibronectin (purple).

Head and neck squamous cell carcinoma SCC61 cells express endogenous MT1-MMP (Figure 1A). Invadopodia's ECM-degrading activity was evaluated by incubating the cells on glass coated with Dylight 633-labeled fibronectin over cross-linked gelatin. ECM degradation was observed as dark spots in the fluorescent ECM layer (Figure 1B). Invadopodia are visualized as actin puncta located in the ECM degradation spots. As previously reported, ECM degradation indeed depends on expression of MT1-MMP, as knockdown of MT1-MMP, using different shRNA sequences, also abolished the invadopodia-mediated ECM degradation (Figure 1B and C). Therefore, we employed this cell line to analyze MT1-MMP localization and turnover at invadopodia.

To visualize live-dynamics of MT1-MMP on the plasma membrane, we employed phLuorin-tagged MT1-MMP (MT1-Luo). The phLuorin, a pH-sensitive modified GFP, enabled us to monitor MT1-MMP exposed on the plasma membrane separately from that in transport vesicles, based on the different pH values in the compartments [22]. MT1-Luo was stably expressed in SCC61 cells, and those cells, expressing it at a similar level to that of the endogenous MT1-MMP, were used for experiments. MT1-MMP represented by MT1-Luo indeed co-localized to invadopodia (Figure 1D). Fibronectin was labeled with Dylight 633, actin with Rhodamine-phalloidin. When the degradation spots were observed closer in the picture, it was of particular interest to note that MT1-Luo co-localized with actin as well, and that they appear to be surrounding degradation spots (Figure 1D).

**Figure 2:**
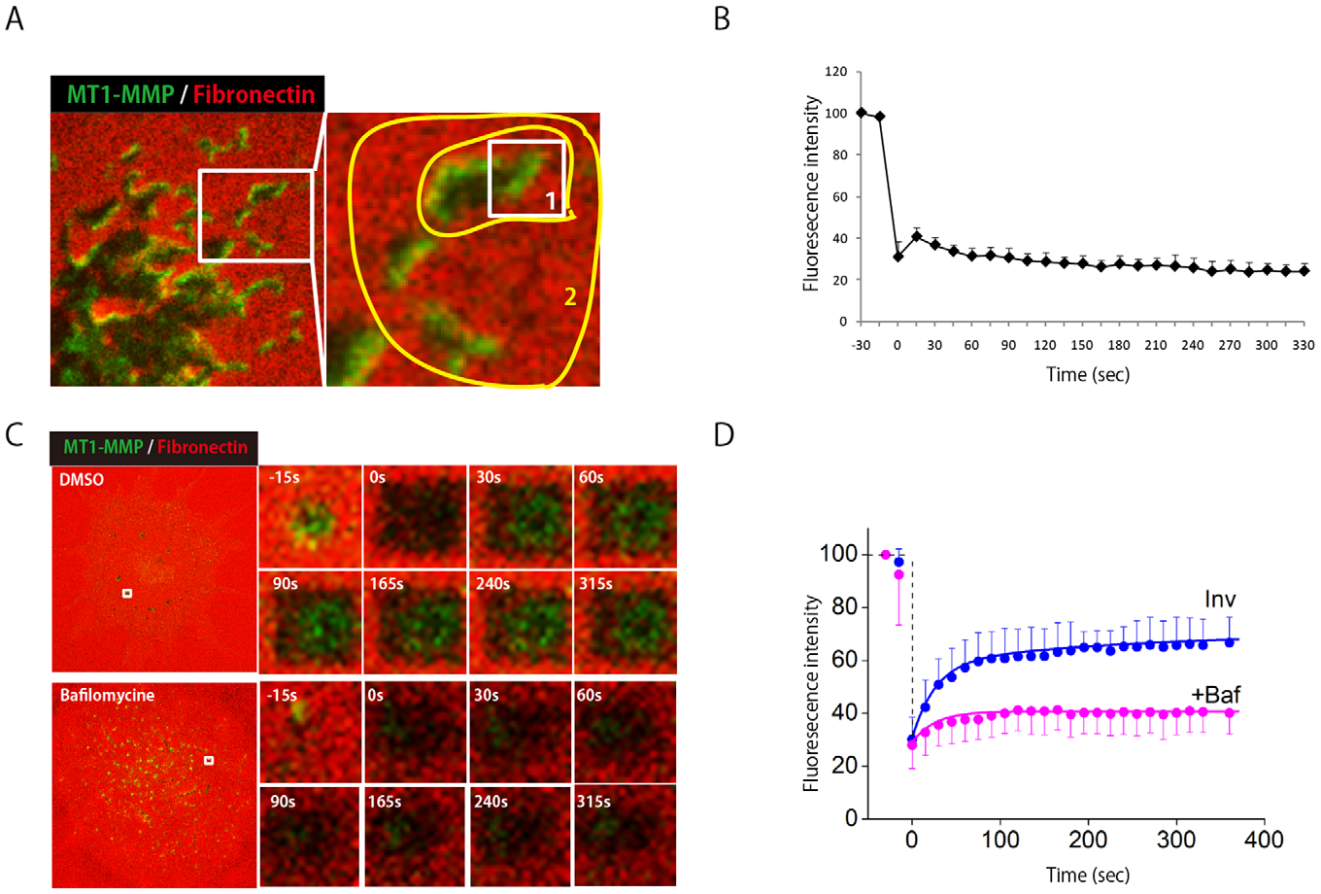
MT1-MMP transport to invadopodia via lysosomal secretion. (A) MT1-MMP-phLuorin-expressing SCC61 cells cultured on Dylight 633-labeled fibronectin were subjected to FRAP- continuous photobleaching experiments. One half of the invadopodia area, indicated by the open box area in region 1, was the FRAP experimental area. Region 2 was a continuous photobleaching area. (B) Quantification of fluorescence recovery in the Figure 2A FRAP region is calculated. (C) Representative images of FRAP experiments at invadopodia without or with bafilomycin. (D) The recovery of FRAP signals are shown in the absence (blue circles) and in the presence (pink circles) of bafilomycin. Reconstructed time courses of fluorescence recovery in the absence (blue line) and in the presence (pink line)of bafilomycin at invadopodia are also shown. The reconstructed FRAP signals show a good agreement with experimental data.

MT1-MMP is thought to be supplied to invadopodia continuously by exocytosis. In order to test various transport pathways to invadopodia, we used bafilomycin A1, which inhibits lysosome function by inhibiting vacuolar-type H(+)-ATPase (V-ATPase) and thereby inhibiting the lysosomal vesicle transport pathway; brefeldin A, which inhibits transport of proteins from ER to the Golgi system; and dynasore, which inhibits dynamin function and thereby prevents both endocytosis and trans-Golgi exit [23]. When cells were treated for 18 hours, all of these inhibitors suppressed ECM degradation (Figure S1 in Text S1). Bafilomycin inhibited expansion of the ECM degradation spots almost immediately, accompanied by increased visibility of intracellular vesicles because of H increase (Figure 2C and D). These results suggest that MT1-MMP is supplied to the invadopodia mainly by recycling mediated by lysosomes rather than by direct exocytosis from the Golgi apparatus to the invadopodia.

### Invadopodia form distinct membrane area with limited mobility

The above data indicate that continuous transport of MT1-MMP into the plasma membrane is critical for maintenance of the invadopodia's ECM-degrading activity. However, it is unclear to what extent lateral diffusion of MT1-MMP contributes to localization of the protease at invadopodia. To evaluate this possibility, we monitored fluorescence recovery after photo-bleaching (FRAP) of MT1-Luo at invadopodia. One-half of an invadopodium area, indicated by the open box area in region 1 in Figure 2A, was photo-bleached. To prevent a supply of MT1-Luo from the cytoplasm to reach the photobleached area by vesicle transport, we continuously bleached the surrounding area (region 2) (Figure 2A). Most of the cytoplasmic vesicles have to travel across the bleached “region 2” when they try to reach the invadopodium. If the membrane mobility of MT1-MMP at invadopodia were sufficient to allow lateral diffusion of MT1-Luo, the bleached area would be refilled by MT1-Luo from the non-bleached area of the same invadopodium. Our results indicated that there was no fluorescence recovery in the photobleached area in region 1 during the indicated time period in Figure 2B. Taken together, these results indicate that lateral diffusion of MT1-Luo within invadopodia is almost negligible.

### Lysosomal secretion regulates quick recycling of MT1-MMP to invadopodia

Since lateral diffusion is almost negligible in invadopodia, we then used FRAP to analyze the kinetics of vesicle transport to invadopodia. Photo-bleaching of MT1-Luo at invadopodia was 60% at 60 sec after having decreased once to 30% of the control level, but the recovery did not reach 100% even after 330 sec; instead, it approached a plateau at around 65% (Figure 2D, blue circles). We observed that FRAP at invadopodia was abrogated by bafilomycin (Figure 2D, +Baf, pink circles). Thus, quick insertion of MT1-Luo into the membrane of invadopodia is mostly mediated by a bafilomycin-sensitive pathway (lysosomal pathway).

### Analysis of FRAP experiments revealed two independent recovery processes at invadopodia

To determine the time constant of fluorescence recovery at invadopodia, we redrew experimental data in a semi-logarithmic scale (Figure S2 in Text S1). The recovery was approximated by a double exponential plot with fast and slow time constants of 26.0 and 259 s, and with the contributions (amplitudes) of 40.7% and 17.5%respectively. The recovery was not complete and reached an asymptotic level of 41.8%. After the application of bafilomycin, however, the recovery was approximated by a single time constant of 49.0 s. Thus, the slow recovery was not observed during the bafilomycin treatment. These results indicate the existence of two independent recovery processes for MT1-MMPs at the surface of invadopodia, and suggest that the slow recovery process is sensitive to bafilomycin, but the fast one is insensitive to it.

### Reconstruction of FRAP experiments

**Figure 3:**
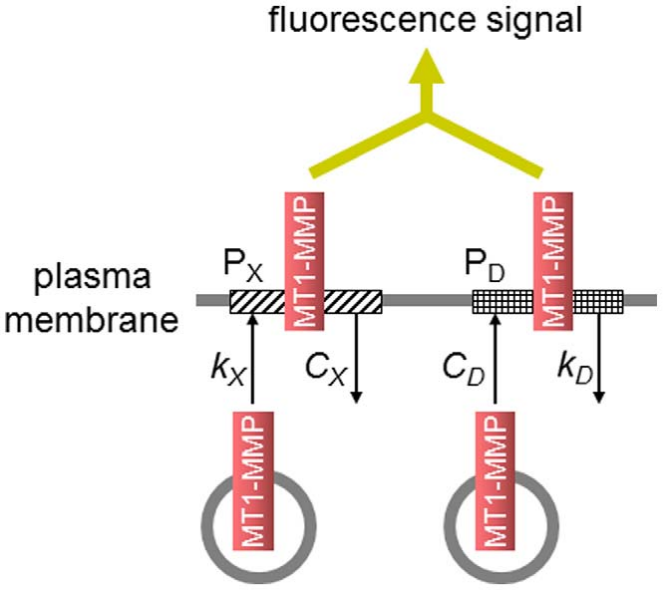
Schematically illustrated model for a rapid turnover. Two pools of MT1-MMP, pool X (P_X_) and pool D (P_D_), were assumed. In pool X, insertion was dependent on the surface density of MT1-MMP, and internalization proceeds at a constant rate. While in pool D, insertion proceeds at a constant rate, and internalization was dependent on the surface density.

From our experiments and analyses we found that the recovery of MT1-MMP at invadopodia is composed of two independent processes. Since the contribution of lateral diffusion of MT1-MMP within invadopodia was negligible (Figure 2A and B), these processes are most simply modeled by two surface density-dependent internalizations of MT1-MMP. However, the slow process is sensitive to bafilomycin, which blocked the supply of MT1-MMP from the lysosomal pathway in our experiments. Therefore, we constructed a model represented by one bafilomycin-sensitive insertion process and one surface density-dependent internalization process. Thus the model is composed of two pools, X and D of MT1-MMP at invadopodia with the recovery time constants of 259 s and 26.0 s, respectively (Figure 3). The observed fluorescence signal is the sum of the signals from pools X and D. In pool X, insertion of MT1-MMP depends on the surface density of MT1-MMP, which was suggested from our experiments (see below), and internalization takes place at a constant rate. In contrast, in pool D, insertion takes place at a constant rate, and internalization depends on the surface density of MT1-MMP.

Our experiment showed that the invadopodial area occupied by MT1-MMP before bleaching was not refilled completely even at the saturated level of fluorescence recovery. On the contrary, the newly inserted MT1-MMP resided at a different area in addition to the area occupied before bleaching. In addition, the invadopodial area occupied by MT1-MMP did not cover the whole invadopodial area at any time during the recovery. Thus, there seemed to be a limited number of sites available for MT1-MMP insertion in invadopodia (Figure S3 in Text S1). Although the underlying mechanism for occupying the limited number of sites at invadopodia is currently not known, this assumption is consistent with our experiments. Therefore, we assume that there is a limited number of sites (or docking sites) for MT1-MMP in pool X. Transported MT1-MMP can be inserted only at free sites on the membrane.

We derived the following equation for the surface density of MT1-MMP at invadopodia:
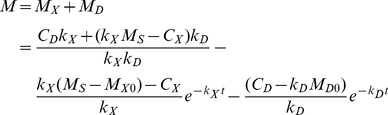
(1)where *M* is the total amount of MT1-MMP at the region of interest at an invadopodium, which is the sum of the amounts in pool D (*M_D_*) and pool X (*M_X_*) and corresponds to the fluorescence intensity before photobleaching. *C_D_* (*k_D_*) and *k_X_* (*C_X_*) are insertion (internalization) rate constant for pools D and X, respectively. *M_S_* is the saturated amount of MT1-MMP in pool D, and *M_D0_* and *M_X0_* are the amounts of unbleached MT1-MMP in pools X and D, respectively, just after the photobleaching, corresponding to the fluorescence intensity at *t = 0*. Derivation of Equation 1 and the parameter values for Eq.1 are shown in Text S1 in detail.

The reconstructed time courses for fluorescence recovery after photobleaching in the absence and presence of bafilomycin are shown by continuous curves in Figure 2D. The addition of bafilomycin was simulated by setting *k_X_* at zero. The reconstructed FRAP signals using Equation 1 represent quite good agreements with experimental observations. Thus Eq.1 successfully reproduced the observations of FRAP experiments. However, the fit was not complete in the presence of bafilomycin, because, in the experiments, the time constant of the fast recovery became large (from 26.0s in the absence of bafilomycin to 49.0 s in the presence of it) for unknown reasons, which were not considered in the present model.

### Construction of a biochemical reaction model for the degradation of ECM

**Figure 4:**
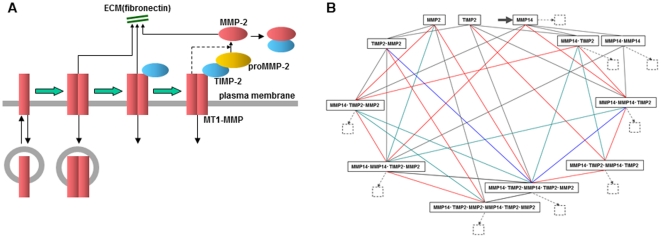
Outline of the model. (A) MT-MMPs in the membrane are dimerized, bound with TIMP-2, and activate proMMP-2. Any MT1-MMP, which is TIMP-2-free, was assumed to degrade ECM. MT1-MMP and all of complexes were assumed to be internalized. (B) Complete diagram of interaction between MT1-MMP, TIMP-2 and proMMP-2. Insertion is shown by a thick arrow for MT1-MMP, and internalization of MT1-MMP is shown in broken-lined arrows with internalized species at small squares. MT1-MMP, TIP-2 and proMMP-2 are designated as M14, T2 and M2 for simplicity.

We next constructed a model for the activation of MMP-2 and the degradation of ECM by MT1-MMP and MMP-2. On the surface of the invadopodial membrane, MT1-MMP is dimerized, bound to TIMP-2, and forms a quadruple complex by binding proMMP-2 (MT1-MMP.MT1-MMP.TIMP-2.MMP-2) as shown in Figure 4A. proMMP-2 in this complex is processed by TIMP-2-free MT1-MMP releasing the active form of MMP-2. Active MMP-2 is inactivated by the binding of TIMP-2.

The state transition diagram of MT1-MMP is shown in Figure 4B. Here, the insertion (thick arrow) and internalization (broken-line arrows) of MT1-MMP are also shown. All MT1-MMP, both TIMP-2-bound and TIMP-2-free, is assumed to be internalized (small squares). The amount of internalized MT1-MMP was balanced by the inserted amount. The insertion to pool D and internalization from pool X were modeled by first order reaction kinetics, while the internalization from pool D and insertion to pool X were modeled as constant processes. The turnover of MT1-MMP by vesicle transport implies the discrete insertion and internalization processes. If these discrete processes are averaged over space and time, however, repetitive insertion and internalization can reasonably be assumed to be continuous processes as in the present model. A full set of A-Cell model and parameter values are shown in Text S1. A simpler state transition diagram was proposed in a previous report [24]. However, there is a possibility that multiple pathways could contribute to the formation and dissociation of a complex, and we could find no reason to avoid some of them. In fact, high multimer complexes such as M14D.M14D.T2.M2 and M14D.T2.M2.M14D.T2.M2 were formed shortly after the start of simulations (Figure S4 in Text S1). Therefore, we employed the full set of possible state transitions for MT1-MMP as shown in Figure 4B.

### Simulation has shown the critical role of MT1-MMP turnover for the degradation of ECM at invadopodia

In the absence of TIMP-2 the quadruple complex cannot be formed, and in excess TIMP-2, the concentration of TIMP-2-free MT1-MMP is reduced and proMMP-2 processing will be reduced significantly. In both cases ECM degradation by activated MMP-2 will be reduced. The concentration of the MMP-2-ECM complex, which is a measure of ECM degradation activity by MMP-2, was maximum at a TIMP-2 concentration of 180 nM, and at both higher and lower concentrations, it was steeply decreased as was expected (Figure S5A in Text S1). In contrast, the concentration of the MT1-MMP-ECM complex was maximum in the absence of TMP-2, and it decreased gradually as the TIMP-2 concentration increased (Figure S5B in Text S1). Since the concentration of the MMP-2-ECM complex decreased very steeply, simulation of ECM degradation was run at a TIMP-2 concentration of 180 nM.

**Figure 5:**
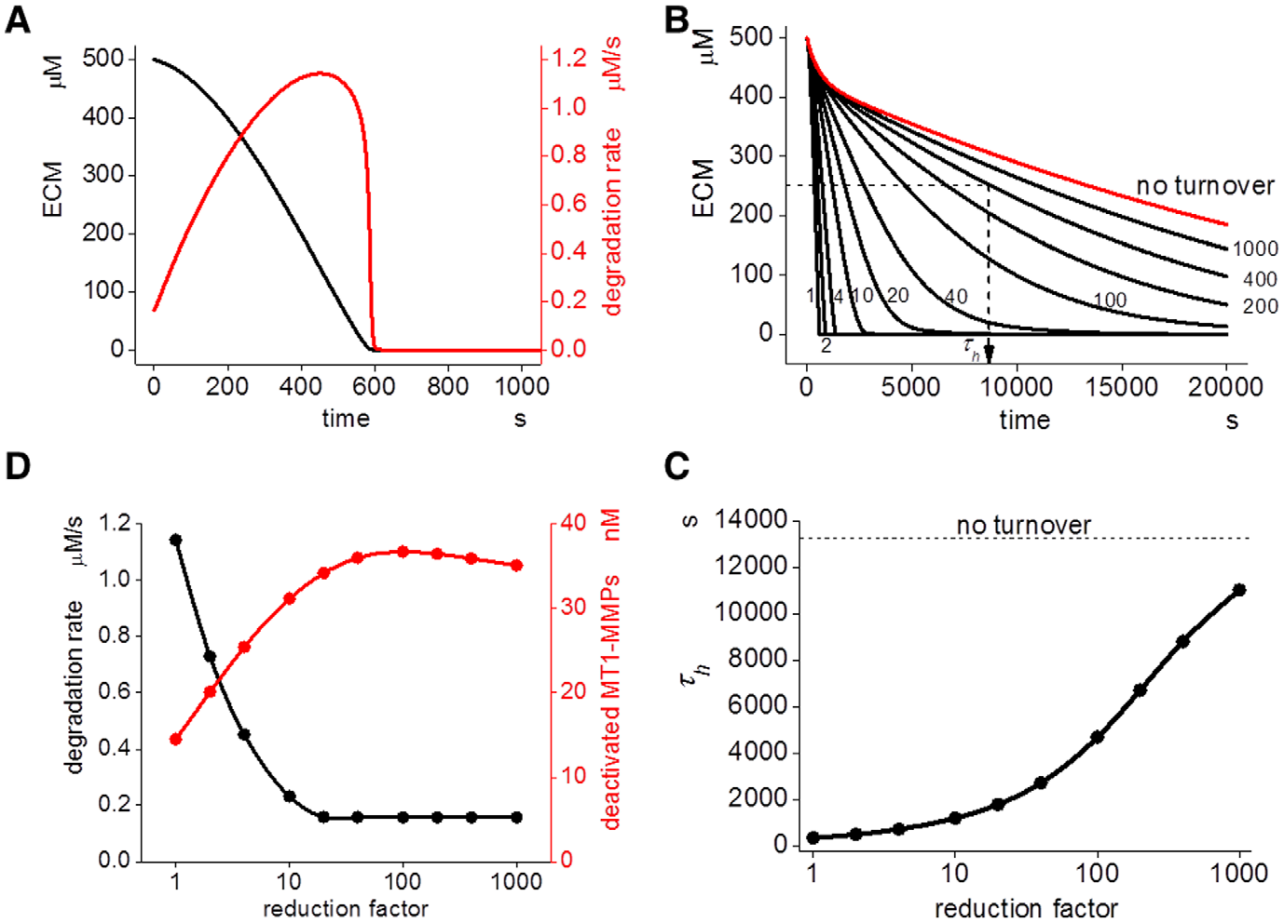
Simulation results of ECM degradation. (A) Simulated ECM degradation with the initial TIMP-2 concentration at 180 nM is shown as a black line. Degradation rate, which is the rate of degradation in µM/s, is shown in red. (B) The time course of ECM degradation with several turnover intervals. If the turnover interval is increased by the factors shown by small numbers, a longer time is required for the same degree of degradation, reaching the state of no turnover (red curve). *τ_h_* is the time required to reach 50% degradation. (C) *τ_h_* increases concomitantly with an increase in the reduction factor. There is a rapid increase in *τ_h_* when the reduction factor is increased tenfold or more. (D) Concomitantly with the increase in reduction factor, the degradation rate (black) quickly decreases and stays almost unchanged at reduction factor increases larger than tenfold. In contrast, the concentration of inactivated MT1-MMP complexes, which cannot degrade ECM or activate proMMP-2, is increased and reaches a plateau level around a reduction factor of 10.

Figure 5A shows time courses of ECM degradation (black) and ECM-degradation rate (red), which is the amount of ECM- degradation per unit time (µM/s). This is the sum of the degradation rates by MT1-MMP and MMP-2 (i.e. k_fn11p_*[M14_X_.fn]+k_fn2p_*[M2_act_.fn]+….. in the model). After the start of ECM degradation, it is completely degraded at 600 s in the control condition, where we used a turnover rate obtained from the experiments.

If we changed the turnover rate of MT1-MMP by changing the insertion (*k_X_*) and internalization (*k_D_*) time constants for pools X and D, while keeping the surface concentration of MT1-MMP and its complex constant, the degradation rate was changed. As shown in Figure 5B, the ECM degradation became slow as the reduction factor (small numbers) approached the state of no turnover. A reduction factor of ten indicates one-tenth values of both *k_X_* and *k_D_* at the same time.

Let the time to reach 50% degradation of ECM (*τ_h_*) be a measure of the rate of ECM degradation. Smaller *τ_h_* values indicate a higher rate of degradation. There was little change in *τ_h_* when the reduction factor was below ten (Figure 5C). When it was higher than ten, however, *τ_h_* increased considerably together with the increase in the reduction factor. Thus the rate of ECM-degradation decreased nonlinearly by the decrease in the turnover rate of MT1-MMP. This indicates that the turnover rate of MT1-MMP is a critical regulator of the ECM-degrading efficacy. The sensitivity analysis, shown in Figure S6, indicated also that the turnover rate of MT1-MMP is one of the sensitive parameters affecting *τ_h_*.

Next we sought the reason for the reduced ECM-degradation that was associated with the reduced turnover rate of MT1-MMP. We found that the ECM-degradation rate at a time point corresponding to 50% ECM degradation was decreased by a value corresponding to the increase in the reduction factor (Figure 5D). In contrast, the concentration of inactivated MT1-MMPs—which is the sum of the TIMP-2-occupied MT1-MMP species such as MT1-MMP-TIMP-2, MT1-MMP-TIMP2-MT1-MMP-TIMP2, or MT1-MMP-TIMP-2-MMP-2—increased concomitantly with the increase in the reduction factor. Thus the decrease in the ECM degradation activity, which was caused by the increase in the TIMP-2-occupied and inactivated MT1-MMP, caused the increase in *τ_h_* by an increase in the turnover interval.

### Rapid turnover is essential to the effective degradation of ECM both in experiments and spatiotemporal simulation

Activated MMP-2 is diluted by diffusion after it is released from the MT1-MMP complex, and diffusing TIMP-2, which replenishes free TIMP-2, increases the inhibition of MT1-MMP at the site of its inhibition by TIMP-2. All these effects reduce ECM degradation in a three-dimensional (3D) space. To visualize these possibilities, we ran spatiotemporal simulations of ECM degradation (Figure S7 in Text S1). The extracellular space was divided into small compartments allowing us to simulate reaction-diffusion (see Text S1for diffusion coefficient).

**Figure 6:**
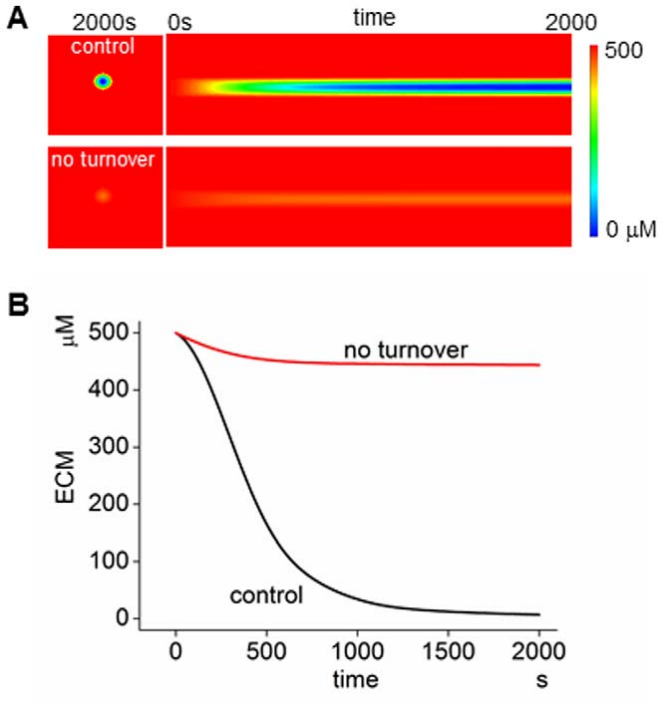
Simulation results for spatiotemporal model. (A) Simulated degradation of ECM. With rapid turnover, MT1-MMP causes degradation of ECM (top panel), while in its absence, no appreciable ECM degradation is seen (bottom panel). (B) The time course of ECM degradation in the absence and presence of the turnover of MT1-MMP. The importance of the turnover for the effective degradation of ECM is clearly seen.

In the case with the MT1-MMP turnover observed in our experiments, the ECM was greatly degraded at 800 s (upper panel of Figure 6A). In the case of no turnover, however, no appreciable ECM degradation was seen (lower panel of Figure 6A). This is clearly seen by the time course shown in Figure 6B. Although a small fraction of ECM was degraded during the first 500 s, no appreciable ECM degradation took place between 500 s and 2,000 s. Thus, rapid turnover of MT1-MMP at invadopodia, as found in our experiments at invadopodia is essential for the effective ECM degradation seen in our simulation. The essential role of rapid MT1-MMP turnover is also shown in experiments, in which no appreciable ECM degradation was seen in the presence of bafilomycin, Dynasore or Brefeldin A (Figure S1 in Text S1).

As shown in Figure S8 in Text S1, the relation between *τ_h_* and the reduction factor in spatiotemporal simulations was comparable with that shown in Figure 5C, and the relation of the degradation rate and inactivated MT1-MMP complexes with the reduction factor was similar to that shown in Figure 5D.

### Simultaneous reduction of the turnover rate and the concentration of MT1-MMP resulted in synergetic reduction in ECM degradation

**Figure 7:**
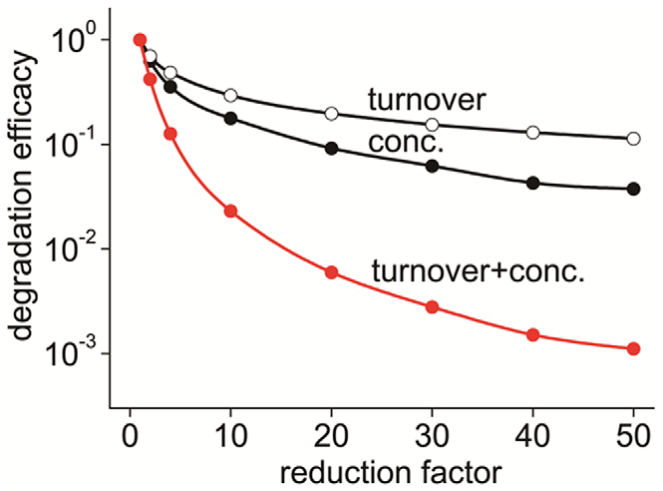
Reduction in the ECM-degradation efficacy by a reduced turnover rate and/or reduced concentration of MT1-MMP. The degradation efficacy is defined as *1/τ_h_* and plotted as normalized values at the control condition (reduction factor = 1). A larger degradation efficacy indicates a faster degradation of ECM. At the same reduction factor, the reduction in degradation efficacy was higher for a reduced concentration (conc.) than for a reduced turnover rate (turnover). If both were reduced simultaneously, however, an unexpectedly larger reduction in the ECM reduction was observed (turnover+conc.).

As a reduction in the concentration of MT1-MMP will also lead to a reduced ECM degradation, we asked what would be more effective for the reduction in ECM degradation, a reduced turnover rate or the concentration of MT1-MMP As shown in Figure 7, we found that the reduction in the MT1-MMP concentration (conc.) was more effective in the reduction of ECM-degradation than a decreased turnover rate (turnover). The degradation efficacy was defined by *1/τ_h_*. It is interesting, however, that if we reduced the turnover rate and the concentration of MT1-MMP at the same time (turnover+conc.), a nonlinearly large and synergetic reduction in the degradation efficacy was observed. While the separate reductions of the turnover rate and the concentration of MT1-MMP to a reduction factor of 10 resulted in the reduced degradation efficacies of 29.4% and 17.7%, respectively, the simultaneous reduction resulted in the degradation efficacy of 2.30% of the control value.

## Discussion

Here we have revealed a rapid turnover of the surface MT1-MMP with time constants of 26.0 and 259 s at invadopodia by experiments. In addition, we have demonstrated both by experiments and simulations that ECM degradation was blocked by blocking of the rapid turnover of MT1-MMP. Thus we have revealed a critical role of the rapid turnover for the effective ECM degradation at invadopodia (Figure S9 in Text S1). Furthermore, we have shown in simulations that simultaneous reductions in the turnover rate and the vesicular content of MT1-MMP reduced the ECM degradation synergistically by simulations. It has been reported that invadopodia-associated actin comets represented a biphasic recovery in FRAP signals: the initial fast recovery (15–20 s) followed by a second slow recovery which reached a plateau level at ∼3 min [25]. It will be of interest to compare these time constants with those for the FRAP signal in the present report.

ECM degradation at invadopodia provides room for initial protrusions. Further degradation provides additional space for the elongation and enlargement of invadopodia. These processes are thought to finally lead to the invasion of cancer cells [6], [26], [27]. There is a clear evidence for the involvement of MT1-MMP in these processes [7], [8], [13]. On the other hand, MT1-MMP-containing vesicles trafficking and fusing to the plasma membrane were shown to be important for the ECM degradation [12], and inhibited or decreased ECM degradation by drugs that block vesicle transport has been reported [14], [28], [29]. Our present study presents data supporting the involvement of the rapid turnover of MT1-MMP for the initial stage of invasion.

Since rapid delivery of vesicles carrying MT1-MMP to invadopodia is important for the ECM degrading activity, it is of particular interest how this delivery process is regulated. Bafilomycin inhibits acidification of lysosomes. Therefore, MT1-MMP appears to be immediately delivered to invadopodia via an endosome-lysosome pathway rather than by the Golgi apparatus to the plasma membrane, which is preferentially inhibited by brefeldin. Indeed, Steffen et al. reported that VAMP7, which associates with late endosome-lysosome vesicles and plays a role in membrane fusion, is required for MT1-MMP delivery to invadopodia and regulates invadopodia formation by supporting MT1-MMP-mediated ECM degradation. Colocalization of MT1-MMP-mCherry with GFP-VAMP7 at invadopodia accompanying ECM degrading was demonstrated [14].

The internalization of ErbB receptors (ErbBRs) has been extensively studied, and its rate constant was reported to be in the range of 0.04–0.2/min depending on the ligand-occupancy [30], [31]. In contrast, the recovery of fluorescence signal from MT1-MMP proceeded with time constants of 26.0 and 259 s in our study. From these values, we estimated an internalization rate for pools D of 2.31/min ( = 60 s/26.0 s), which is one-order of magnitude higher than that of ErbBRs. The internalization rate for transferrin receptor was reported to be around 1/min [30], [31]. We have shown a more than twofold higher rate for MT1-MMP. Such a rapid turnover of MT1-MMP at invadopodia is presumably critical for MT1-MMP to degrade ECM because MT1-MMP is inactivated rapidly by TIMP-2 as shown in our simulation results.

Endocytosis of receptors has been recognized as a mechanism for their down-regulation. For example, ErbB receptors are known to be internalized upon ligand binding, and this process down-regulated receptor activity [32]. In the present model, however, the internalization of MT1-MMP in pool X increased the ECM-degrading activity by offering free sites for the insertion of new MT1-MMP onto the membrane surface. Thus the internalization of MT1-MMP can provide the opposite effect to that of ErbBR. If the renewal of the surface MT1-MMPs was blocked, the concentration of inactivated MT1-MMP increases by binding of TIMP-2 as shown in Figures 6D and Figure S8B in Text S1. This blocks both binding of MT1-MMP to ECM proteins and activation of proMMP-2. Both of these effects will lead to a decreased ECM degradation. Therefore, we conclude that the rapid turnover and repetitive insertion of surface MT1-MMP is a basis for the effective ECM degradation at invadopodia.

In our model we assumed the existence of pools X and D with different insertion and internalization mechanisms for MT1 MMP. However, the observed fluorescence recovery with two time constants can be explained by two pools D or two pools X. Nevertheless, our bafilomycin experiments indicated the existence of different mechanism for the two time-constant processes. Therefore we assumed different insertion and internalization mechanisms for pools X and D.

Mathematical models showing cancer cell invasion of tissue have been reported previously [33], [34], [35]. One of the main interests in these models is in the invasive behavior of a population of cells. Our model, as presented in this work, focuses on the invasive behavior of a subcellular structure, invadopodia. These two approaches are different both in spatial and temporal scales. The consolidation of the two approaches will be a challenge that will be approached in the near future.

Here we have found a rapid turnover of surface MT1-MMP at invadopodia with a time constant of 26.0 s. The reduction in the turnover rate according to both fast and slow time constants reduced the rate of ECM degradation as shown in Figures 5 and 6. In addition, with simultaneous reductions in both the turnover rate and the concentration of MT1-MMP, nonlinear and marked reduction in the ECM degradation was seen in our simulations (Figure 7). Thus, the rate of the turnover may be a critical therapeutical parameter in addition to the inhibition of MT1-MMP. The suggested combination treatment derived from our mathematical model may make it possible to inhibit invadopodia-mediated invasion selectively compared to other suggested interventions.

## Materials and Methods

### Cell culture

The SCC61 cell lines have been described previously [10]. Cells were maintained in DMEM with 20% fetal bovine serum with 0.4 mg/ml hydrocortisone.

### Invadopodia assay

The matrix degradation assay was done as described by Chen et al. Briefly, fibronectin (BD Biosciences) was labeled with Dylight 633 (Fisher) by dialysis in borate buffer [0.17 mol/L borate, 0.075 mol/L NaCl (pH 9.3)]. The buffer was changed to PBS and dialyzed extensively for 3 to 4 days. To coat MatTek dishes, 2.5% gelatin/2.5% sucrose in PBS added to the dish, followed by crosslinking with 0.5% glutaraldehyde in PBS. 50 ug/mL solution of fluorescence-labeled fibronectin was incubated with the cross-linked gelatin in MatTek dishes in the dark for 1 h. The dish was sterilized with 70% ethanol, washed with PBS, and equilibrated with invadopodia medium [DMEM supplemented with 15% FBS and 5% Nu-Serum (BD)] for 30 min before the addition of cells. For invadopodia assays, 7×10^4^ cells were suspended in 2 mL of invadopodia medium containing 100 uM EGF and added to the plate for 18 h (parental cells) or 5 h (MT1-Luo cells).

### Confocal photobleaching experiments

A combination of both FRAP and continuous photobleaching techniques was developed on a confocal laser-scanning microscope Nikon A1 microscope using Nice elements Software (Nikon). MT1-Luo cells cultured on Dylight 633 labeled fibronectin for 5 hours were then bleached and scanned at two different regions of interest (Region 1 and 2) using bleaching laser excitation settings. During the bleaching phase, the region 1 (FRAP region) was excited with regular imaging settings, whereas continuous bleaching settings were used at the continuous photobleaching region.

Cells were imaged using an inverted confocal laser-scanning microscope (LSM 510, Carl Zeiss MicroImaging). The 488-nm line of an argon laser was used to excite phLuorin. For all samples, a Zeiss Plan-Neofluar 63X/1.3 oil immersion lens was used for imaging. FRAP experiments were performed at room temperature. For confocal FRAP measurements squares were chosen as ROIs. Within the squares were photobleached for either 100 or 200 scan iterations using 100% transmission of the 488-nm-wavelength laser.

### Construction of computational model

The models were constructed using A-Cell 36,37, which can be downloaded from http://www.ims.u-tokyo.ac.jp/mathcancer/index.html. The models for pools X and D comprise interaction between MT1-MMP with TIMP-2 and formation of ternary complex (MT1-MMP-TIMP-2-MMP-2), internalization and insertion of MT1-MMP, proMMP-2 activation by MT1-MMP, MMP-2 inactivation by TIMP-2, and ECM degradation by MT1-MMP and MMP-2. Biochemical reaction schemes were embedded into the three-dimensional shape, as a model for the degradation of ECM. Details of the model and parameter values are shown in the Table S1 in Text S1, and can be downloaded from the same site as the A-Cell download. In the A-Cell model, MT1-MMP, TIMP-2, and MMP-2 were designated as M14, T2, and M2 for simplicity.

### Simulations

A reaction-diffusion simulation program in C language was automatically generated by A-Cell from the constructed model, and compiled using Intel C++ Studio XE 2011 for Linux. The differential equations were numerically integrated by the fourth-order Runge-Kutta method. Simulations were run on a Linux-based system with Intel Xeon X5680 3.33 GHz.

## Supporting Information

Text S1 Supplement figures S1, S2, S3, S4, S5, S6, S7, S8, S9 with legends, and texts for “Mathematical reconstruction of FRAP signals”, and “Detail of the Model”.
(PDF)
