# Supplementary material for: Establishment and Validation of Computational Model for MT1-MMP Dependent ECM Degradation and Intervention Strategies
Source: PLoS Comput Biol. 2012 Apr 12;8(4):e1002479. doi: 10.1371/journal.pcbi.1002479 (PMC3325185; doi:10.1371/journal.pcbi.1002479)
Supplement: Text S1 — Supplement figures S1, S2, S3, S4, S5, S6, S7, S8, S9 with legends, and texts for “Mathematical reconstruction of FRAP signals”, and “Detail of the Model”. (PDF) [file pcbi.1002479.s001.pdf]

Text S1

Supplementary information for “Establishment and validation of computational model for MT1-MMP dependent ECM degradation and intervention strategies”

Daisuke Hoshino, Naohiko Koshikawa, Takashi Suzuki, Vito Quaranta,
Alissa M. Weaver, Motoharu Seiki and Kazuhisa Ichikawa

Supplementary Figures

A

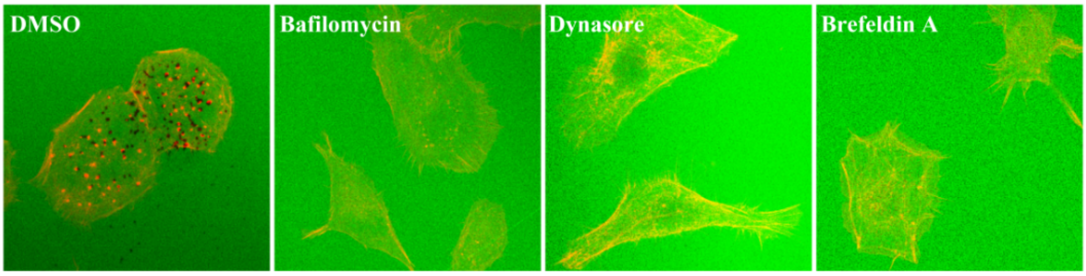

B

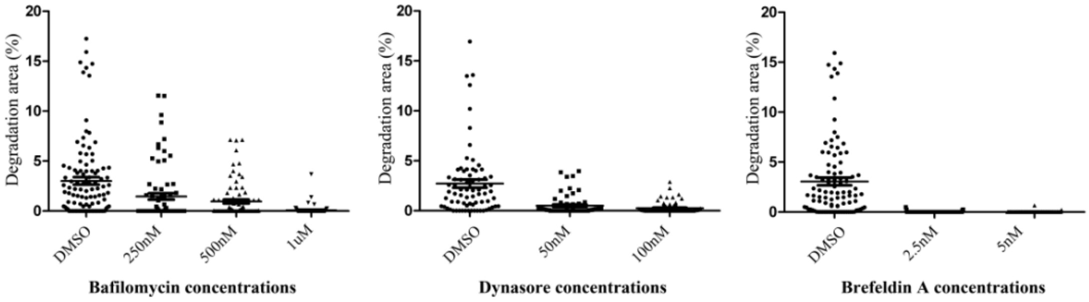

Figure S1.

Effects of inhibitors of exocytic/endocytic pathways on the ECM degradation. Treatment of the cells with either Bafilomycin A1, which disrupts lysosome function; Dynasore, which inhibits dynamin function; or Brefeldin A, which inhibits transport of proteins from ER to Golgi; inhibited invadopodia-mediated ECM degradation in a dose-dependent manner (B). Representative pictures are displayed in A.

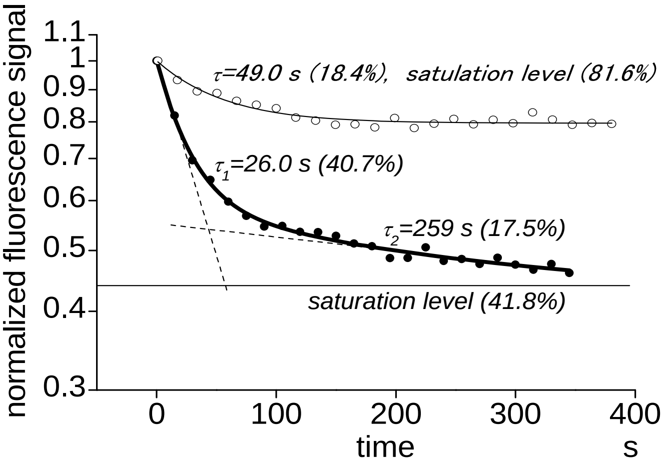

Figure S2.

To determine the time constant of fluorescence recovery at invadopodia, we redrew the experimental data in a semi-logarithmic scale. In the control invadopodia (closed circles for experiments), the recovery was approximated by a double exponential plot with fast and slow time constants of 26.0 and 259 s, and with the contributions (amplitudes) of 40.7% and 17.5%, respectively (the thick line, R^2=0.996). The recovery was not complete and reached an asymptotic level of 41.8%. By the application of bafilomycin (open circles for experiments), however, the recovery was approximated by a single time constant of 49.0 s (the thin line, R^2=0.946).

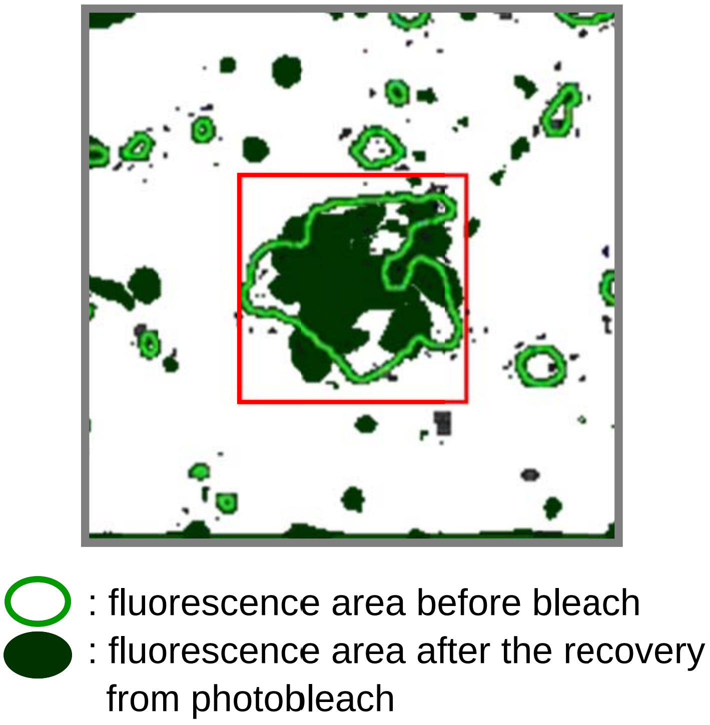

Figure S3.

Fluorescence area before the photobleaching and after the complete recovery. We have analyzed the image taken of an invadopodium as shown here. The red square indicates the area of photobleaching, which covered a single invadopodium. Before photobleaching, fluorescence was detected from areas surrounded by the light green lines. But after the recovery from the photobleaching, fluorescence came from dark green areas. Note that the fluorescent areas before and after the photobleachig did not overlap completely. In some regions fluorescence was detected only before photobleaching, but not after the recovery, while in other regions, fluorescence was detected only after photobleaching. These two areas did not overlap completely. This suggests the existence of a limited number of “sites” for MT1-MMP at the surface of invadopodia. Available (free) sites before and after the photobleaching did not coincide completely, and new MT1-MMPs inserted after photobleaching seemed to be inserted only to the available sites at the time of insertion. Note that the area occupied by MT1-MMP throughout the observation was not the whole invadopodial area throughout the observation of a single invadopodium.

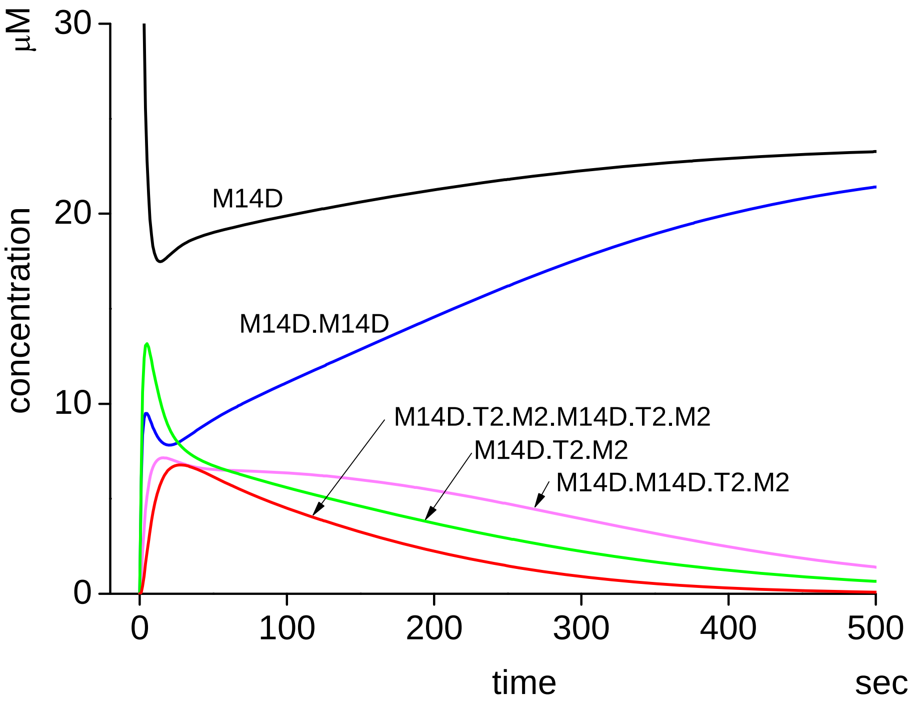

Figure S4.

The time course of formation of complexes in the present model. It is evident that high multimer complexes such as M14D.M14D.T2.M2 and M14D.T2.M2.M14D.T2.M2 are formed at the very beginning of the simulation, thus affecting the time course of formation of species in the model.

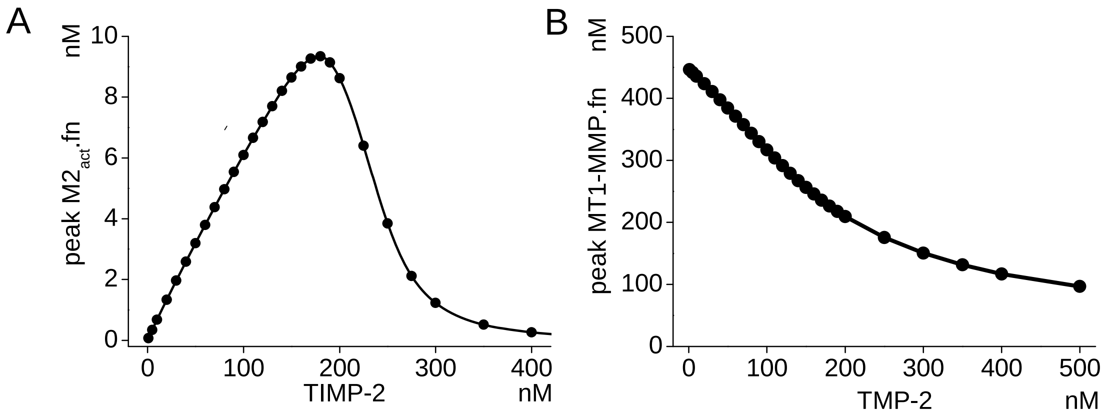

Figure S5.

The concentrations of activated MMP-2-ECM (A) and MT1-MMP-ECM complexes (B) are measures of ECM degradation. The MMP-2-ECM complex has a single peak at a TIMP-2 concentration of 180 nM as was expected, and both at lower and higher TIMP-2 concentrations the concentration of the complex was decreased. The MT1-MMP-ECM complex decreased gradually by the increase in TIMP-2.

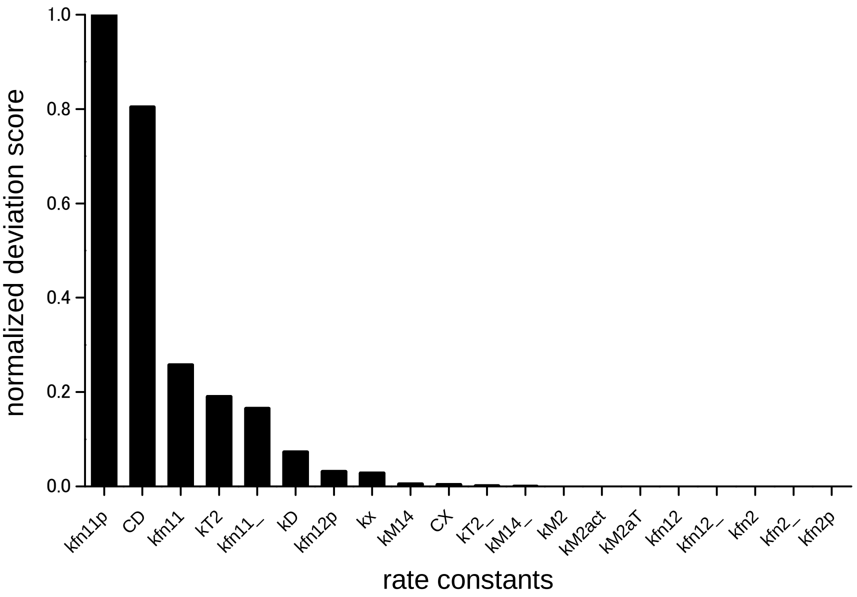

Figure S6.

Sensitivity analysis. We performed sensitivity analyses based on the single-variable variation method as was reported before (Joo et al, 2007). The normalized score of deviation from the reference value of τ_h is plotted against 20 rate constants. Rate constants for ECM degradation such as kfn11, kfn11_ and kfn11p are sensitive parameters as was expected. It is important to note that the turnover rates of surface MT1-MMP such as CD, kD and kx are also sensitive parameters. This indicates that a change in these parameters alters τ_h significantly (see main text).

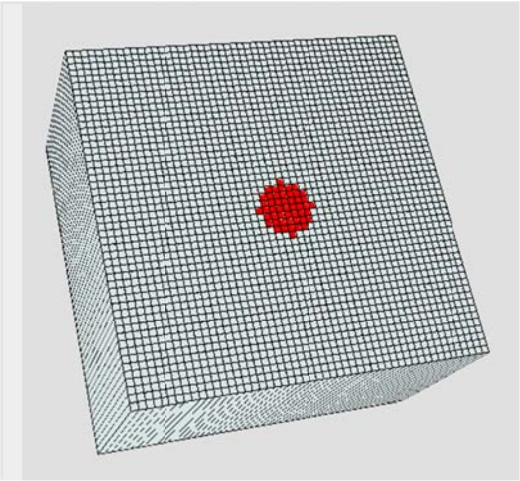

Figure S7.

Three-dimensional shape was cuboid with 5 μm (W) x 5 μm (D) and 3 μm (H). The shape was divided into 51x51x1 compartments. Thus the size of each compartment was 0.0973 μm x 0.0973 μm x 3 μm. One surface of the shape was assumed to be the ventral surface of cancer cells and ECM was present all along the shape. We set 49 compartments at the center of the shape as an invadopodium (shown in red) whose diameter was about 0.9 μm.

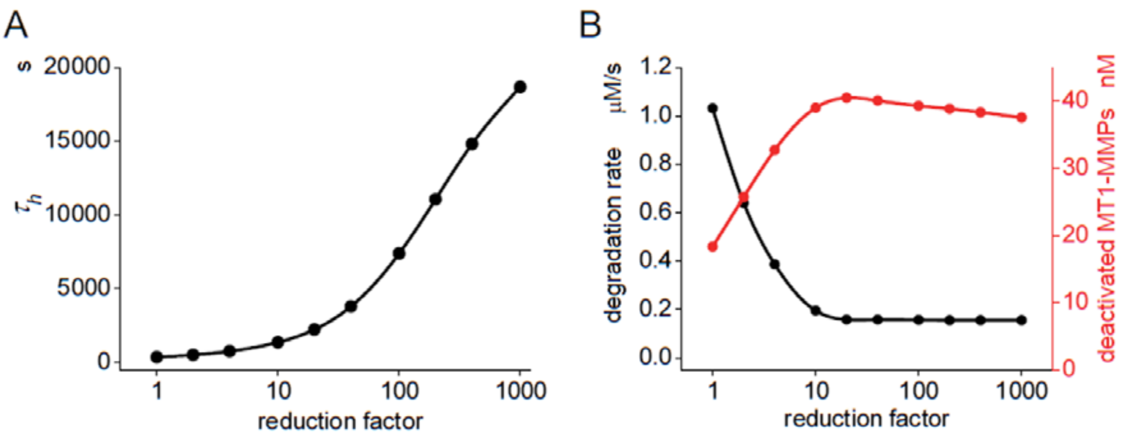

Figure S8.

The changes in τ_h (A) and the degradation activity (black line and symbols) and the concentration of inactivated MT1-MMP complexes (red line and symbols) (B) in spatiotemporal model. The change in τ_h as a function of the reduction factor is shown in Figure S8A. The changes in the degradation rate (black) and the concentration of inactivated MT1-MMP complexes (red) by the change in the reduction factor in the spatiotemporal model are shown in Figure S8B. All of these curves are almost the same as those of the point model shown in Figures 5C and D, indicating that the reason for the much decreased ECM-degradation efficacy seen in the case of reduced turnover rate is the same as in the point model.

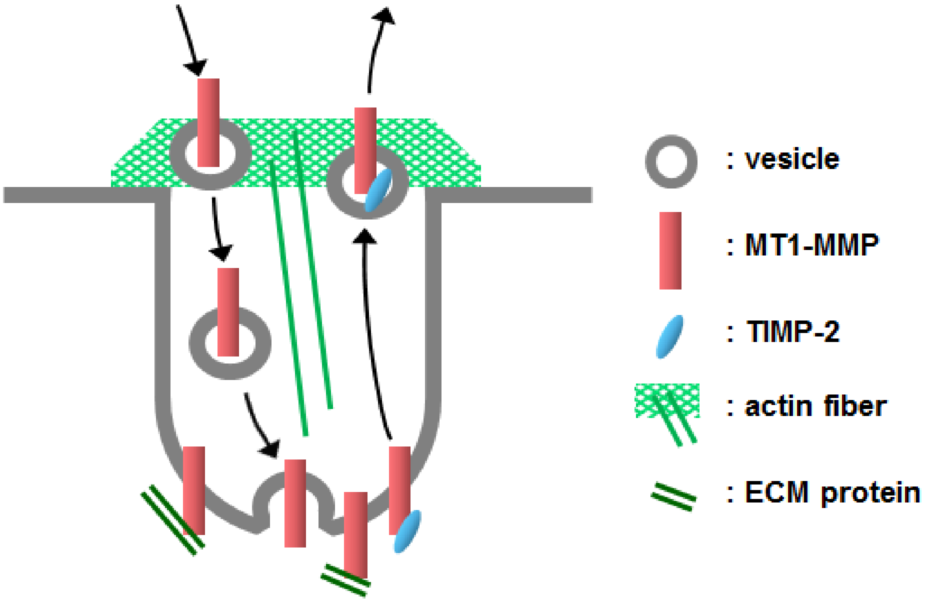

Figure S9.

Schematical drawing of the turnover of surface MT1-MMP at invadopodia. There is a constant turnover of MT1-MMP at invadopodia, accomplished by the continuous insertion to and internalization from the surface of invadopodia. This enables the continuous renewal of TIMP-2-free and ECM-degrading forms of MT1-MMP at the surface of invadopodia.

Mathematical reconstruction of FRAP signals

By the analysis of fluorescence recovery at invadopodia, we found two time constants in the exponential recovery process (see main text). This indicates that the recovery was composed of two independent processes with different time constants. We assume the existence of pools X and D with different recovery time constants in the invadopodial membrane, where MT1-MMP recovery proceeds independently in the two pools. The measured fluorescence was the sum of that from pools X and D. In pool X, insertion depends on the available sites on the invadopodial membrane, which was suggested as shown in Figure S3 (see also main text). We assumed a first order reaction rate constant k_X for insertion. The internalization for pool X is a constant process with the rate C_X. In contrast, in pool D, insertion is a constant process with the rate C_D and the internalization depends on the surface density of MT1-MMP with the first order reaction rate constant k_D. Insertion to pool X was assumed to be inhibited by bafilomycin, while for pool D, insertion was assumed to be insensitive to bafilomycin.

For pools X and D, we get the following differential equation for the insertion and internalization processes.

$$dM_X/dt = k_X(M_S - M_X) - C_X, \qquad \text{S1)}$$

$$dM_D/dt = C_D - k_D M_D, \qquad \text{S2)}$$

where M_X and M_D are the surface concentration of MT1-MMP, and M_S is the saturated concentration of MT1-MMP on the surface of invadopodia. Thus M_S - M_X gives the available “sites” for the insertion of MT1-MMP.

With initial conditions at $t=0$ of $M_X=M_{X0}$ and $M_D=M_{D0}$, we get the following analytical solutions for Eqs S1 and S2:

$$M_X = \frac{(k_X M_S - C_X) - (k_X(M_S - M_{X0}) - C_X)e^{-k_X t}}{k_X} \ , \qquad \text{S3)}$$

$$M_D = \frac{C_D - (C_D - k_D M_{D0})e^{-k_D t}}{k_D} \ . \qquad \text{S4)}$$

By combining Eqs. S3 and S4, we can calculate the total MT1-MMP concentration on an invadopodium as follows:

$$M = M_X + M_D$$

$$= \frac{C_D k_X + (k_X M_S - C_X)k_D}{k_X k_D} - \frac{k_X(M_S - M_{X0}) - C_X}{k_X} e^{-k_X t} - \frac{(C_D - k_D M_{D0})}{k_D} e^{-k_D t}. \qquad \text{S5)}$$

The fluorescence signal measured in our experiments should be proportional to M.

To get parameter values in Eq. S5, we compared Eq. S5 with the equation obtained from the experiment and its analysis. As shown in Figure S10, experimental data was transformed from y to Y

by transformation of y→y'→y''→Y for the double exponential curve fitting.

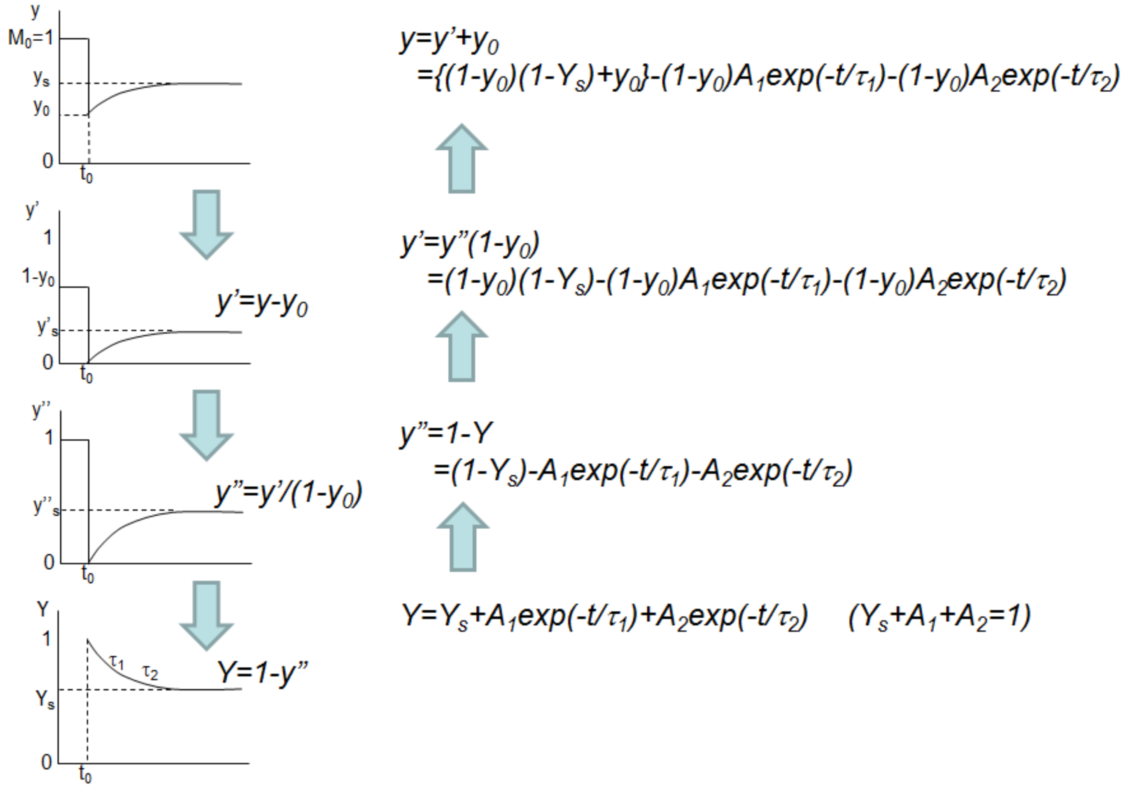

Figure S10. Transformation and inverse transformation of experimental data.

The top graph is a schematical drawing of the fluorescence signal observed in experiments. This graph is finally transformed to the bottom one for the exponential fitting, where we get the equation shown on the right. Then we execute the inverse transformation to get an equation shown on the top and below:

$$y = \left((1-y_0)(1-Y_S)+y_0\right) - (1-y_0)A_1e^{-t/\tau_1} - (1-y_0)A_2e^{-t/\tau_2}. \qquad \text{S6)}$$

Y_S, A_1, A_2, τ_1, and τ_2 are directly derived from the curve fitting, and we obtained $Y_S=0.419$, $A_1=0.407$, $A_2=0.174$, $\tau_1=26.0$ s, and $\tau_2=259$ s. y_0 is directly obtained from the graph, which is 0.306. By comparing Eqs. S5 and S6 assuming that A_1 (A_2) and τ_1 (τ_2) correspond to pool D (X), we get the following relation.

$$\left.\begin{aligned}
&\frac{C_Dk_X+(k_XM_S-C_X)k_D}{k_Xk_D} = (1-y_0)(1-Y_S)+y_0 = 0.709,\\
&\frac{C_D-k_DM_{D0}}{k_D} = (1-y_0)A_1 = 0.282,\\
&\frac{k_X(M_S-M_{X0})-C_X}{k_X} = (1-y_0)A_2 = 0.121,\\
&k_D = 1/26\ /\text{s}, \qquad k_X=1/259\ /\text{s}.
\end{aligned}\right\} \text{S7)}$$

From Eq. S7, the following set of relations was derived.

$$\left.\begin{array}{l} M_S = 0.709 - \frac{C_D}{k_D} + \frac{C_X}{k_X}\ , \\ M_{D0} = \frac{C_D}{k_D} - 0.282, \\ M_{X0} = M_S - 0.121 - \frac{C_X}{k_X}, \\ k_D = 1/26\ /\mathrm{s}, \qquad k_X = 1/259\ /\mathrm{s}. \end{array}\right\} \qquad \text{S8)}$$

From the equilibrium condition at a sufficiently large *t*, the following relation should follow:

$$C_X = k_X\left(M_S - M_{X\infty}\right), \qquad C_D = k_D M_{D\infty}, \qquad \text{S9)}$$

where $M_{X\infty}$ and $M_{D\infty}$ are concentration of MT1-MMP at a sufficiently large time after photobleaching.

At $t=0$, the following relation should follow:

$$\frac{M_{X0}}{M_{D0}} = \frac{A_2}{A_1} = 0.430. \qquad \text{S10)}$$

On the other hand, from Eq. S5 the following relation should follow:

$$M_{X\infty} + M_{D\infty} = \frac{C_D k_X + (k_X M_S - C_X) k_D}{k_X k_D}. \qquad \text{S11)}$$

In addition, we assume the following:

$$M_S = 0.43 M_0 = 0.43. \qquad \text{S12)}$$

where M_o is the concentration of MT1-MMP before photobleaching.

From Eqs.8-12) we get

$$M_{X0}=9.18\mathrm{x}10^{-2}, \qquad M_{D0}=2.14\mathrm{x}10^{-1},$$
$$M_{X\infty}=2.13\mathrm{x}10^{-1}, \qquad M_{D\infty}=4.96\mathrm{x}10^{-1}. \qquad \text{S13)}$$

If we assume $M_0=141$ nM, we get the following values from Eqs. S9 and S13.

M_S=60.6 nM,

M_{X0}=12.9 nM, M_{D0}=30.1 nM,

$M_{X\infty}$=30.1 nM, $M_{D\infty}$=69.9 nM, S14)

C_X=1.18x10^{-10} M/s, C_D=2.69x10^{-9} M/s.

Now we have all parameter values for Eq. S5.

Detail of the model

All theoretically available complexes and all possible interactions between them are shown in Figure 5B. There are several pathways to reach each complex. These pathways were modeled by A-Cell as shown in the group “complexes of MT1-MMP with TIMP-2 and MMP-2 for PX” of Figure S11 for pool X. MT1-MMP is designated by M14 in short with suffix “x”. TIMP-2 and proMMP-2 are designated by T2 and M2 in short too.

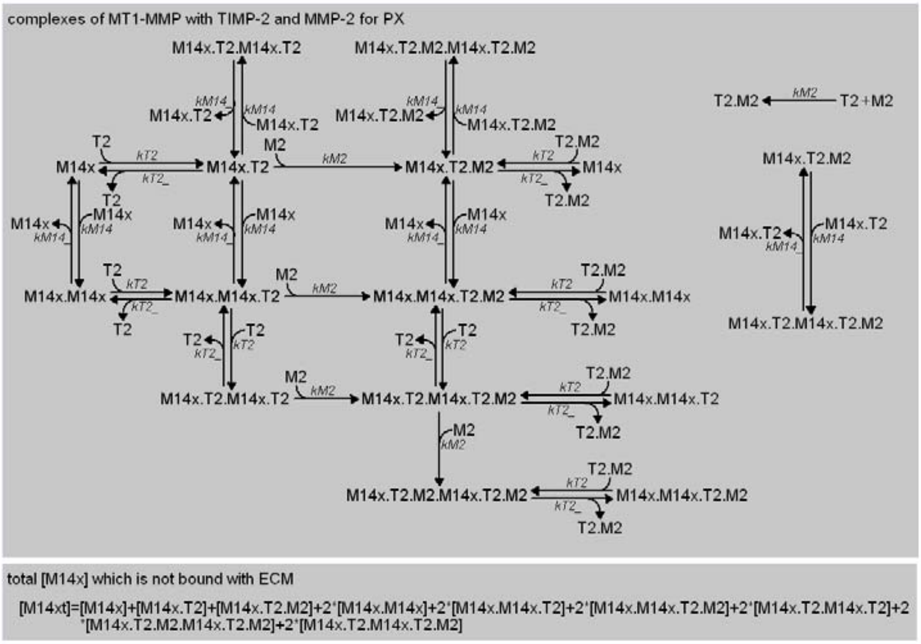

Figure S11. A-Cell model for the state transition of MT1-MMP. Here we show only the model for pool X. The scheme was the same for pool D.

The group “total [M14x] which is not bound with ECM” describes an equation for the total amount of MT1-MMP unbound to ECM. There is the same scheme for pool D.

Turnover of MT1-MMP for pools X and D was modeled as shown in Figure S12. Insertion was modeled by a simple first-order reaction (Cf. groups “insertion of MT1-MMP to PX” and “insertion of MT1-MMP to PD”). For pool X, insertion is dependent on the available sites, MF, and it is calculated by the group “calculation of free sites for PX”, where M14xGT is the total concentration of MT1-MMP. Internalization of MT1-MMP in pool D is dependent on the surface MT1-MMP concentration. Then it is modeled by simple first-order reactions as shown in the group “internalization of MT1-MMP and recycling of TIMP2 and MMP2 for PD”. Monomeric MT1-MMP in addition to all complexes of MT1-MMPs was assumed to be internalized. TIMP-2 and proMMP-2 are assumed to be recycled.

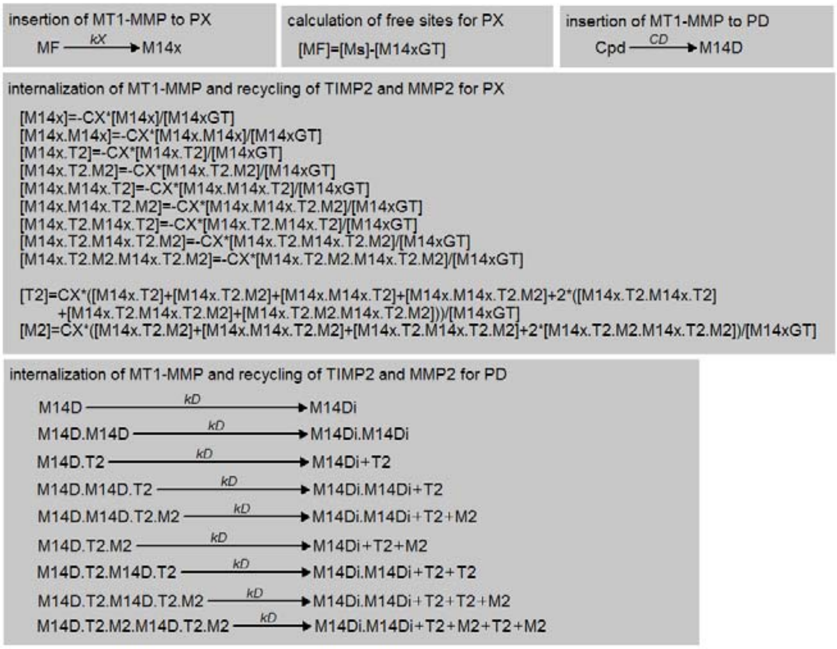

Figure S12. A-Cell model for the turnover of MT1-MMP.

Internalization of MT1-MMP at pool X is described by differential equations as shown in the group "internalization of MT1-MMP and recycling of TIMP2 and MMP2 for PX". Since the rate of internalization of each complex should be proportional to the relative total MT1-MMP concentration, and this is impossible to describe by a first-order reaction, we described the internalization for pool X using differential equations.

The activation and inactivation of MMP-2 are modeled as shown in the groups "MMP2 activation" and "MMP2 inactivation" in Figure S13. There are four groups for which to calculate total concentrations of several species. M14a is the total concentration of MT1-MMP that can degrade ECM. Dimers free from TIMP-2 were assumed to have a twofold higher activity than the single molecules for ECM degradation. M14ma is the total concentration that can activate proMMP-2.

M14xGT (M14DGT) is the total MT1-MMP concentration in pool X (pool D) including the ECM-bound form. M2xt (M2Dt) and T2xt (T2Dt) are the total MMP-2 and TIMP-2 concentrations of the MT1-MMP-bound form in pool X (pool D), which are used for the calculation of total MMP-2 and TIMP-2 concentration (M2t and T2t in the group "total MT1-MMP, TIMP2 and MMP2 concentration". M14t is the total MT1-MMP concentration.

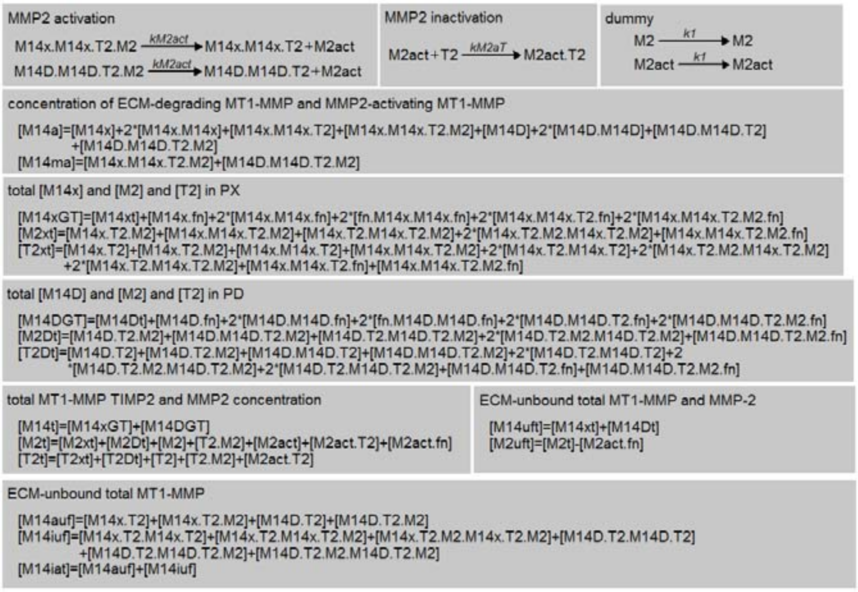

Figure S13. A-Cell model for the MMP-2 activation, inactivation, and calculation of total concentration.

ECM Degradation by MT1-MMP and active MMP-2 was assumed to follow Michaelis-Menten kinetics. The reaction schemes are shown in the groups “ECM degradation by MT1-MMP” and “ECM-degradation by MMP-2” of Figure S14. M14cmplx in the group “ECM-degradation complex by MT1-MMP” is the total concentration of ECM-bound form of MT1-MMP.

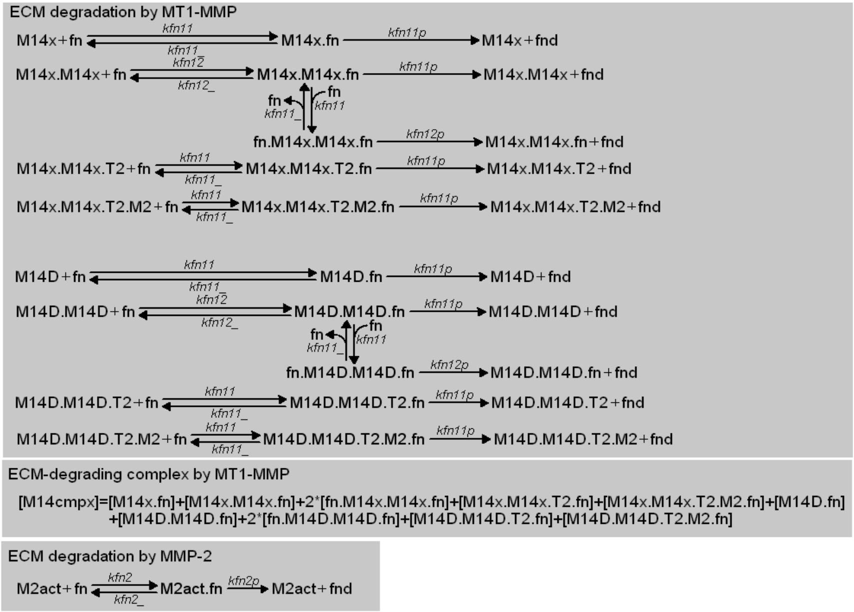

Figure S14. A-Cell model for the degradation of ECM by MT1-MMP and by active MMP-2.

The full set of differential equations automatically derived from the above A-Cell model are shown below.

/* MT1-MMP TIMP2 MMP2 ternary complex for PX */

d[M14x]/dt = -kT2[M14x]*[T2] +kT2_*[M14x.T2] -kM14*[M14x]*[M14x] +kM14_*[M14x.M14x]*
-kM14[M14x]*[M14x] +kM14_*[M14x.M14x] -kM14*[M14x.T2]*[M14x]*
+kM14_*[M14x.M14x.T2] -kM14[M14x.T2.M2]*[M14x] +kM14_*[M14x.M14x.T2.M2]*
-kT2[M14x]*[T2.M2] +kT2_*[M14x.T2.M2] -CX*[M14x]/[M14xGT] +kX*[MF]*
-kfn11[M14x]*[fn] +kfn11_*[M14x.fn] +kfn11p*[M14x.fn]*

d[T2]/dt = -kT2[M14x]*[T2] +kT2_*[M14x.T2] -kT2*[M14x.M14x]*[T2] +kT2_*[M14x.M14x.T2]*
-kT2[M14x.M14x.T2]*[T2] +kT2_*[M14x.T2.M14x.T2] -kT2*[M14x.M14x.T2.M2]*[T2]*
+kT2_*[M14x.T2.M14x.T2.M2] -kM2[T2]*[M2] -kT2*[M14D]*[T2] +kT2_*[M14D.T2]*
-kT2[M14D.M14D]*[T2] +kT2_*[M14D.M14D.T2] -kT2*[M14D.M14D.T2]*[T2]*
+kT2_*[M14D.T2.M14D.T2] -kT2[M14D.M14D.T2.M2]*[T2]*
+kT2_*[M14D.T2.M14D.T2.M2] -kM2[T2]*[M2] +CX*([M14x.T2] +[M14x.T2.M2]*
+[M14x.M14x.T2] +[M14x.M14x.T2.M2] +2([M14x.T2.M14x.T2] +[M14x.T2.M14x.T2.M2]*
+[M14x.T2.M2.M14x.T2.M2]))/[M14xGT] +kD[M14D.M14D.T2] +kD*[M14D.M14D.T2.M2]*
+kD[M14D.T2.M2] +kD*[M14D.T2.M14D.T2] +kD*[M14D.T2.M14D.T2]*
+kD[M14D.T2.M14D.T2.M2] +kD*[M14D.T2.M14D.T2.M2] +kD*[M14D.T2]*
+kD[M14D.T2.M2.M14D.T2.M2] +kD*[M14D.T2.M2.M14D.T2.M2] -kM2aT*[M2act]*[T2]*

d[M14x.T2]/dt = kT2[M14x]*[T2] -kT2_*[M14x.T2] -kM14*[M14x.T2]*[M14x] +kM14_*[M14x.M14x.T2]*
-kM14[M14x.T2]*[M14x.T2] +kM14_*[M14x.T2.M14x.T2] -kM14*[M14x.T2]*[M14x.T2]*
+kM14_*[M14x.T2.M14x.T2] -kM14[M14x.T2.M2]*[M14x.T2]*
+kM14_*[M14x.T2.M14x.T2.M2] -kM2[M14x.T2]*[M2] -CX*[M14x.T2]/[M14xGT]*

d[M14x.M14x]/dt = kM14[M14x]*[M14x] -kM14_*[M14x.M14x] -kT2*[M14x.M14x]*[T2]*
+kT2_*[M14x.M14x.T2] -kT2[M14x.M14x]*[T2.M2] +kT2_*[M14x.M14x.T2.M2]*
-CX[M14x.M14x]/[M14xGT] -kfn12*[M14x.M14x]*[fn] +kfn12_*[M14x.M14x.fn]*
+kfn11p[M14x.M14x.fn]*

d[M14x.M14x.T2]/dt = kT2[M14x.M14x]*[T2] -kT2_*[M14x.M14x.T2] +kM14*[M14x.T2]*[M14x]*
-kM14_*[M14x.M14x.T2] -kT2[M14x.M14x.T2]*[T2] +kT2_*[M14x.T2.M14x.T2]*
-kT2[M14x.M14x.T2]*[T2.M2] +kT2_*[M14x.T2.M14x.T2.M2] -kM2*[M14x.M14x.T2]*[M2]*
-CX[M14x.M14x.T2]/[M14xGT] +kM2act*[M14x.M14x.T2.M2] -kfn11*[M14x.M14x.T2]*[fn]*
+kfn11_*[M14x.M14x.T2.fn] +kfn11p[M14x.M14x.T2.fn]*

d[M14x.T2.M2]/dt = -kM14[M14x.T2.M2]*[M14x] +kM14_*[M14x.M14x.T2.M2]*
-kM14[M14x.T2.M2]*[M14x.T2.M2] +kM14_*[M14x.T2.M2.M14x.T2.M2]*
-kM14[M14x.T2.M2]*[M14x.T2.M2] +kM14_*[M14x.T2.M2.M14x.T2.M2]*
+kT2[M14x]*[T2.M2] -kT2_*[M14x.T2.M2] -kM14*[M14x.T2.M2]*[M14x.T2]*

+kM14_*[M14x.T2.M14x.T2.M2] +kM2[M14x.T2]*[M2] -CX*[M14x.T2.M2]/[M14xGT]*

d[M14x.M14x.T2.M2]/dt = kM14[M14x.T2.M2]*[M14x] -kM14_*[M14x.M14x.T2.M2]*

-kT2[M14x.M14x.T2.M2]*[T2] +kT2_*[M14x.T2.M14x.T2.M2]*

+kT2[M14x.M14x]*[T2.M2] -kT2_*[M14x.M14x.T2.M2]*

-kT2[M14x.M14x.T2.M2]*[T2.M2] +kT2_*[M14x.T2.M2.M14x.T2.M2]*

+kM2[M14x.M14x.T2]*[M2] -CX*[M14x.M14x.T2.M2]/[M14xGT]*

-kM2act[M14x.M14x.T2.M2] -kfn11*[M14x.M14x.T2.M2]*[fn]*

+kfn11_*[M14x.M14x.T2.M2.fn]+kfn11p[M14x.M14x.T2.M2.fn]*

d[M14x.T2.M14x.T2]/dt = kT2[M14x.M14x.T2]*[T2] -kT2_*[M14x.T2.M14x.T2]*

+kM14[M14x.T2]*[M14x.T2] -kM14_*[M14x.T2.M14x.T2] -kM2*[M14x.T2.M14x.T2]*[M2]*

-CX[M14x.T2.M14x.T2]/[M14xGT]*

d[M14x.T2.M14x.T2.M2]/dt = kT2[M14x.M14x.T2.M2]*[T2] -kT2_*[M14x.T2.M14x.T2.M2]*

+kT2[M14x.M14x.T2]*[T2.M2] -kT2_*[M14x.T2.M14x.T2.M2]*

+kM14[M14x.T2.M2]*[M14x.T2] -kM14_*[M14x.T2.M14x.T2.M2]*

+kM2[M14x.T2.M14x.T2]*[M2] -kM2*[M14x.T2.M14x.T2.M2]*[M2]*

-CX[M14x.T2.M14x.T2.M2]/[M14xGT]*

d[M14x.T2.M2.M14x.T2.M2]/dt = kM14[M14x.T2.M2]*[M14x.T2.M2] -kM14_*[M14x.T2.M2.M14x.T2.M2]*

+kT2[M14x.M14x.T2.M2]*[T2.M2] -kT2_*[M14x.T2.M2.M14x.T2.M2]*

+kM2[M14x.T2.M14x.T2.M2]*[M2] -CX*[M14x.T2.M2.M14x.T2.M2]/[M14xGT]*

d[T2.M2]/dt = -kT2[M14x]*[T2.M2] +kT2_*[M14x.T2.M2] -kT2*[M14x.M14x]*[T2.M2]*

+kT2_*[M14x.M14x.T2.M2] -kT2[M14x.M14x.T2]*[T2.M2]*

+kT2_*[M14x.T2.M14x.T2.M2] -kT2[M14x.M14x.T2.M2]*[T2.M2]*

+kT2_*[M14x.T2.M2.M14x.T2.M2] +kM2[T2]*[M2]-kT2*[M14D]*[T2.M2]*

+kT2_*[M14D.T2.M2] -kT2[M14D.M14D]*[T2.M2] +kT2_*[M14D.M14D.T2.M2]*

-kT2[M14D.M14D.T2]*[T2.M2] +kT2_*[M14D.T2.M14D.T2.M2]*

-kT2[M14D.M14D.T2.M2]*[T2.M2] +kT2_*[M14D.T2.M2.M14D.T2.M2]*

+kM2[T2]*[M2]*

d[M2]/dt = -kM2[T2]*[M2] -kM2*[M14x.T2]*[M2] -kM2*[M14x.M14x.T2]*[M2]*

-kM2[M14x.T2.M14x.T2]*[M2] -kM2*[M14x.T2.M14x.T2.M2]*[M2] -kM2*[T2]*[M2]*

-kM2[M14D.T2]*[M2] -kM2*[M14D.M14D.T2]*[M2] -kM2*[M14D.T2.M14D.T2]*[M2]*

-kM2[M14D.T2.M14D.T2.M2]*[M2] +CX*([M14x.T2.M2] +[M14x.M14x.T2.M2]*

+[M14x.T2.M14x.T2.M2] +2[M14x.T2.M2.M14x.T2.M2])/[M14xGT]*

+kD[M14D.M14D.T2.M2] +kD*[M14D.T2.M2] +kD*[M14D.T2.M14D.T2.M2]*

+kD[M14D.T2.M2.M14D.T2.M2] +kD*[M14D.T2.M2.M14D.T2.M2] -k1*[M2]+k1*[M2]*

/* total [M14x] which is not bound with ECM */

[M14xt] = [M14x] +[M14x.T2] +[M14x.T2.M2] +2[M14x.M14x] +2*[M14x.M14x.T2]*

+2[M14x.M14x.T2.M2] +2*[M14x.T2.M14x.T2] +2*[M14x.T2.M2.M14x.T2.M2]*

+2[M14x.T2.M14x.T2.M2]*

/* MT1-MMP TIMP2 MMP2 ternary complex for PD */

d[M14D]/dt = -kT2[M14D]*[T2] +kT2_*[M14D.T2] -kM14*[M14D]*[M14D] +kM14_*[M14D.M14D]*

-kM14[M14D]*[M14D] +kM14_*[M14D.M14D] -kM14*[M14D.T2]*[M14D]*

+kM14_*[M14D.M14D.T2] -kM14[M14D.T2.M2]*[M14D] +kM14_*[M14D.M14D.T2.M2]*

-kT2[M14D]*[T2.M2] +kT2_*[M14D.T2.M2] +CD*[Cpd] -kD*[M14D] -kfn11*[M14D]*[fn]*

+kfn11_*[M14D.fn] +kfn11p[M14D.fn]*

d[M14D.T2]/dt = kT2[M14D]*[T2] -kT2_*[M14D.T2] -kM14*[M14D.T2]*[M14D]*

+kM14_*[M14D.M14D.T2] -kM14[M14D.T2]*[M14D.T2] +kM14_*[M14D.T2.M14D.T2]*

-kM14[M14D.T2]*[M14D.T2] +kM14_*[M14D.T2.M14D.T2] -kM14*[M14D.T2.M2]*[M14D.T2]*

+kM14_*[M14D.T2.M14D.T2.M2] -kM2[M14D.T2]*[M2]-kD*[M14D.T2]*

d[M14D.M14D]/dt = kM14[M14D]*[M14D] -kM14_*[M14D.M14D] -kT2*[M14D.M14D]*[T2]*

+kT2_*[M14D.M14D.T2] -kT2[M14D.M14D]*[T2.M2] +kT2_*[M14D.M14D.T2.M2]*

-kD[M14D.M14D] -kfn12*[M14D.M14D]*[fn] +kfn12_*[M14D.M14D.fn]*

+kfn11p[M14D.M14D.fn]*

d[M14D.M14D.T2]/dt = kT2[M14D.M14D]*[T2] -kT2_*[M14D.M14D.T2] +kM14*[M14D.T2]*[M14D]*

-kM14_*[M14D.M14D.T2] -kT2[M14D.M14D.T2]*[T2] +kT2_*[M14D.T2.M14D.T2]*

-kT2[M14D.M14D.T2]*[T2.M2] +kT2_*[M14D.T2.M14D.T2.M2]*

-kM2[M14D.M14D.T2]*[M2] -kD*[M14D.M14D.T2] +kM2act*[M14D.M14D.T2.M2]*

-kfn11[M14D.M14D.T2]*[fn] +kfn11_*[M14D.M14D.T2.fn] +kfn11p*[M14D.M14D.T2.fn]*

d[M14D.T2.M2]/dt = -kM14[M14D.T2.M2]*[M14D] +kM14_*[M14D.M14D.T2.M2]*

-kM14[M14D.T2.M2]*[M14D.T2.M2] +kM14_*[M14D.T2.M2.M14D.T2.M2]*

-kM14[M14D.T2.M2]*[M14D.T2.M2] +kM14_*[M14D.T2.M2.M14D.T2.M2]*

+kT2[M14D]*[T2.M2] -kT2_*[M14D.T2.M2] -kM14*[M14D.T2.M2]*[M14D.T2]*

+kM14_*[M14D.T2.M14D.T2.M2] +kM2[M14D.T2]*[M2] -kD*[M14D.T2.M2]*

d[M14D.M14D.T2.M2]/dt = kM14[M14D.T2.M2]*[M14D] -kM14_*[M14D.M14D.T2.M2]*

-kT2[M14D.M14D.T2.M2]*[T2] +kT2_*[M14D.T2.M14D.T2.M2] +kT2*[M14D.M14D]*[T2.M2]*

-kT2_*[M14D.M14D.T2.M2] -kT2[M14D.M14D.T2.M2]*[T2.M2] +kT2_*[M14D.T2.M2.M14D.T2.M2]*

+kM2[M14D.M14D.T2]*[M2] -kD*[M14D.M14D.T2.M2] -kM2act*[M14D.M14D.T2.M2]*

-kfn11[M14D.M14D.T2.M2]*[fn] +kfn11_*[M14D.M14D.T2.M2.fn] +kfn11p*[M14D.M14D.T2.M2.fn]*

d[M14D.T2.M14D.T2]/dt = kT2[M14D.M14D.T2]*[T2] -kT2_*[M14D.T2.M14D.T2]*

+kM14[M14D.T2]*[M14D.T2] -kM14_*[M14D.T2.M14D.T2] -kM2*[M14D.T2.M14D.T2]*[M2]*

-kD[M14D.T2.M14D.T2]*

d[M14D.T2.M14D.T2.M2]/dt = kT2[M14D.M14D.T2.M2]*[T2] -kT2_*[M14D.T2.M14D.T2.M2]*

+kT2[M14D.M14D.T2]*[T2.M2] -kT2_*[M14D.T2.M14D.T2.M2]*

+kM14*[M14D.T2.M2]*[M14D.T2] -kM14_*[M14D.T2.M14D.T2.M2]

+kM2*[M14D.T2.M14D.T2]*[M2] -kM2*[M14D.T2.M14D.T2.M2]*[M2]

-kD*[M14D.T2.M14D.T2.M2]

d[M14D.T2.M2.M14D.T2.M2]/dt = kM14*[M14D.T2.M2]*[M14D.T2.M2]

-kM14_*[M14D.T2.M2.M14D.T2.M2] +kT2*[M14D.M14D.T2.M2]*[T2.M2]

-kT2_*[M14D.T2.M2.M14D.T2.M2] +kM2*[M14D.T2.M14D.T2.M2]*[M2]

-kD*[M14D.T2.M2.M14D.T2.M2]

/* total [M14D] which is not bound with ECM */

[M14Dt] = [M14D]+[M14D.T2] +[M14D.T2.M2] +2*[M14D.M14D] +2*[M14D.M14D.T2]

+2*[M14D.M14D.T2.M2] +2*[M14D.T2.M14D.T2] +2*[M14D.T2.M2.M14D.T2.M2]

+2*[M14D.T2.M14D.T2.M2]

/* insertion of MT1-MMP to PX */

d[MF]/dt = -kX*[MF]

/* calculation of free sites for PX */

[MF]=[Ms]-[M14xGT]

/* internalization of MT1-MMP and recycling of TIMP2 and MMP2 for PD */

d[M14Di]/dt = kD*[M14D]+kD*[M14D.T2.M2]+kD*[M14D.T2]

d[M14Di.M14Di]/dt = kD*[M14D.M14D] +kD*[M14D.M14D.T2] +kD*[M14D.M14D.T2.M2]

+kD*[M14D.T2.M14D.T2] +kD*[M14D.T2.M14D.T2.M2] +kD*[M14D.T2.M2.M14D.T2.M2]

/* concentration of ECM-degrading MT1-MMP and MMP2-activating MT1-MMP */

[M14a]=[M14x]+2*[M14x.M14x]+[M14x.M14x.T2]+[M14x.M14x.T2.M2]+[M14D]+2*[M14D.M14D]

+[M14D.M14D.T2]+[M14D.M14D.T2.M2]

[M14ma]=[M14x.M14x.T2.M2]+[M14D.M14D.T2.M2]

/* total MT1-MMP TIMP2 and MMP2 concentration */

[M14t]=[M14xGT]+[M14DGT]

[M2t]=[M2xt]+[M2Dt]+[M2]+[T2.M2]+[M2act]+[M2act.T2]+[M2act.fn]

[T2t]=[T2xt]+[T2Dt]+[T2]+[T2.M2]+[M2act.T2]

/* MMP2 activation */

d[M2act]/dt = kM2act*[M14x.M14x.T2.M2] +kM2act*[M14D.M14D.T2.M2] -kM2aT*[M2act]*[T2]

-k1*[M2act] +k1*[M2act] -kfn2*[M2act]*[fn] +kfn2_*[M2act.fn] +kfn2p*[M2act.fn]

/* MMP2 inactivation */

d[M2act.T2]/dt = kM2aT*[M2act]*[T2]

/* total [M14x] and [M2] and [T2] in PX */

[M14xGT]=[M14xt]+[M14x.fn]+2*[M14x.M14x.fn]+2*[fn.M14x.M14x.fn]+2*[M14x.M14x.T2.fn]

+2*[M14x.M14x.T2.M2.fn]

[M2xt]=[M14x.T2.M2]+[M14x.M14x.T2.M2]+[M14x.T2.M14x.T2.M2]+2*[M14x.T2.M2.M14x.T2.M2]

+[M14x.M14x.T2.M2.fn]

[T2xt]=[M14x.T2]+[M14x.T2.M2]+[M14x.M14x.T2]+[M14x.M14x.T2.M2]+2[M14x.T2.M14x.T2]*

+2[M14x.T2.M2.M14x.T2.M2]+2*[M14x.T2.M14x.T2.M2]+[M14x.M14x.T2.fn]+[M14x.M14x.T2.M2.fn]*

/* total [M14D] and [M2] and [T2] in PD */

[M14DGT]=[M14Dt]+[M14D.fn]+2[M14D.M14D.fn]+2*[fn.M14D.M14D.fn]+2*[M14D.M14D.T2.fn]*

+2[M14D.M14D.T2.M2.fn]*

[M2Dt]=[M14D.T2.M2]+[M14D.M14D.T2.M2]+[M14D.T2.M14D.T2.M2]+2[M14D.T2.M2.M14D.T2.M2]*

+[M14D.M14D.T2.M2.fn]

[T2Dt]=[M14D.T2]+[M14D.T2.M2]+[M14D.M14D.T2]+[M14D.M14D.T2.M2]+2[M14D.T2.M14D.T2]*

+2[M14D.T2.M2.M14D.T2.M2]+2*[M14D.T2.M14D.T2.M2]+[M14D.M14D.T2.fn]*

+[M14D.M14D.T2.M2.fn]

/* ECM-unbound total MT1-MMP */

[M14auf]=[M14x.T2]+[M14x.T2.M2]+[M14D.T2]+[M14D.T2.M2]

[M14iuf]=[M14x.T2.M14x.T2]+[M14x.T2.M14x.T2.M2]+[M14x.T2.M2.M14x.T2.M2]+[M14D.T2.M14D.T2]

+[M14D.T2.M14D.T2.M2]+[M14D.T2.M2.M14D.T2.M2]

[M14iat]=[M14auf]+[M14iuf]

/* ECM-unbound total MT1-MMP and MMP-2 */

[M14uft]=[M14xt]+[M14Dt]

[M2uft]=[M2t]-[M2act.fn]

/* ECM degradation by MT1-MMP */

d[fn]/dt = -kfn11[M14x]*[fn] +kfn11_*[M14x.fn] -kfn12*[M14x.M14x]*[fn] +kfn12_*[M14x.M14x.fn]*

-kfn11[M14x.M14x.T2]*[fn] +kfn11_*[M14x.M14x.T2.fn] -kfn11*[M14x.M14x.T2.M2]*[fn]*

+kfn11_*[M14x.M14x.T2.M2.fn] -kfn11[M14D]*[fn] +kfn11_*[M14D.fn]*

-kfn12[M14D.M14D]*[fn] +kfn12_*[M14D.M14D.fn] -kfn11*[M14D.M14D.T2]*[fn]*

+kfn11_*[M14D.M14D.T2.fn] -kfn11[M14D.M14D.T2.M2]*[fn] +kfn11_*[M14D.M14D.T2.M2.fn]*

-kfn11[M14x.M14x.fn]*[fn] +kfn11_*[fn.M14x.M14x.fn] -kfn11*[M14D.M14D.fn]*[fn]*

+kfn11_*[fn.M14D.M14D.fn] -kfn2[M2act]*[fn] +kfn2_*[M2act.fn]*

d[M14x.fn]/dt = kfn11[M14x]*[fn]-kfn11_*[M14x.fn]-kfn11p*[M14x.fn]*

d[fnd]/dt = kfn11p[M14x.fn] +kfn11p*[M14x.M14x.fn] +kfn11p*[M14x.M14x.T2.fn]*

+kfn11p[M14x.M14x.T2.M2.fn] +kfn11p*[M14D.fn] +kfn11p*[M14D.M14D.fn]*

+kfn11p[M14D.M14D.T2.fn] +kfn11p*[M14D.M14D.T2.M2.fn] +kfn12p*[fn.M14x.M14x.fn]*

+kfn12p[fn.M14D.M14D.fn] +kfn2p*[M2act.fn]*

d[M14x.M14x.fn]/dt = kfn12[M14x.M14x]*[fn] -kfn12_*[M14x.M14x.fn] -kfn11p*[M14x.M14x.fn]*

-kfn11[M14x.M14x.fn]*[fn] +kfn11_*[fn.M14x.M14x.fn] +kfn12p*[fn.M14x.M14x.fn]*

d[M14x.M14x.T2.fn]/dt = kfn11[M14x.M14x.T2]*[fn]-kfn11_*[M14x.M14x.T2.fn]-kfn11p*[M14x.M14x.T2.fn]*

d[M14x.M14x.T2.M2.fn]/dt = kfn11[M14x.M14x.T2.M2]*[fn] -kfn11_*[M14x.M14x.T2.M2.fn]*

-kfn11p[M14x.M14x.T2.M2.fn]*

d[M14D.fn]/dt = kfn11[M14D]*[fn]-kfn11_*[M14D.fn]-kfn11p*[M14D.fn]*

d[M14D.M14D.fn]/dt = kfn12[M14D.M14D]*[fn] -kfn12_*[M14D.M14D.fn] -kfn11p*[M14D.M14D.fn]*

-kfn11[M14D.M14D.fn]*[fn] +kfn11_*[fn.M14D.M14D.fn] +kfn12p*[fn.M14D.M14D.fn]*

d[M14D.M14D.T2.fn]/dt = kfn11[M14D.M14D.T2]*[fn] -kfn11_*[M14D.M14D.T2.fn]*

-kfn11p[M14D.M14D.T2.fn]*

d[M14D.M14D.T2.M2.fn]/dt = kfn11[M14D.M14D.T2.M2]*[fn] -kfn11_*[M14D.M14D.T2.M2.fn]*

-kfn11p[M14D.M14D.T2.M2.fn]*

d[fn.M14x.M14x.fn]/dt = kfn11[M14x.M14x.fn]*[fn]-kfn11_*[fn.M14x.M14x.fn]-kfn12p*[fn.M14x.M14x.fn]*

d[fn.M14D.M14D.fn]/dt = kfn11[M14D.M14D.fn]*[fn] -kfn11_*[fn.M14D.M14D.fn]*

-kfn12p[fn.M14D.M14D.fn]*

/* ECM degradation by MMP-2 */

d[M2act.fn]/dt = kfn2[M2act]*[fn]-kfn2_*[M2act.fn]-kfn2p*[M2act.fn]*

/* ECM-degrading complex by MT1-MMP */

[M14cmpx]=[M14x.fn] +[M14x.M14x.fn] +2[fn.M14x.M14x.fn] +[M14x.M14x.T2.fn]+[M14x.M14x.T2.M2.fn]*

+[M14D.fn] +[M14D.M14D.fn] +2[fn.M14D.M14D.fn] +[M14D.M14D.T2.fn]*

+[M14D.M14D.T2.M2.fn]

/* ECM degrading activity */

[dECMact]=kfn11p[M14cmpx]+kfn2p*[M2act.fn]*

All parameter values are shown in Table S1. Those that are not listed in this table are zero at the onset of simulations. All rate constants for pools X and D are the same except those for insertion and internalization.

Table S1. Parameter values

parameters	values	unit	comments
Cpd・CD	$2.69x10^{-9}$	/s	from our experiment and its analysis
CX	$1.18x10^{-10}$	M/s	from our experiment and its analysis
M14D	$6.99x10^{-8}$	M	from our experiment and its analysis assuming initial MT1-MMP concentration of 141 nM
M14x	$3.01x10^{-8}$	M	from our experiment and its analysis assuming initial MT1-MMP concentration of 141 nM
M2	$1.00x10^{-7}$	M	assumed
MF	$3.06x10^{-8}$	M	calculated from Ms and occupied sites concentration on the membrane
Ms	$6.06x10^{-8}$	M	from our experiment and its analysis assuming initial MT1-MMP concentration of 141 nM
T2	$1.80x10^{-7}$	M	nominal value. Varied from 1 nM to 1 μM in the simulation
Fn	$1.00x10^{-4}$	M	assumed
kD	0.0385	/s	from our experiment and its analysis
kM14	$2.00x10^{6}$	/M/s	calculated from K_D of 5 nM
kM14_	0.01	/s	calculated from K_D of 5 nM
kM2	$2.10x10^{7}$	/M/s	from Murphy and Willenbrock, 1995
kM2aT	$2.10x10^{7}$	/M/s	from Murphy and Willenbrock, 1995
kM2act	0.02	/s	Karagiannis and Poppel, 2004
kT2	$2.74x10^{6}$	/M/s	from Toth et al., 2000
kT2_	$2.00x10^{-4}$	/s	from Toth et al., 2000
kX	0.00386	/s	from our experiment and its analysis
kfn11	$2.00x10^{6}$	/M/s	calculated by assuming kfn11_ of 2.94 /s and kfn12p of 3.18 /s by Olson et al., 1997
kfn11_	2.94	/s	calculated by assuming kfn11_ of 2e6 /M/s and kfn12p of 3.18 /s by Olson et al., 1997
kfn11p	1.59	/s	kfn12p/2
kfn12	$4.00x10^{6}$	/s	2*kfn11
kfn12_	2.94	/s	=kfn11_
kfn12p	3.18	/s	from Olson et al., 1997 assuming the same value for fibronectin
kfn2	$2.00x10^{6}$	/M/s	calculated assuming K_m of 0.6 μM in Gioia et al., 2007
kfn2_	0.93	/s	from Gioia et al., 2007 assuming the same value for fibronectin
kfn2p	0.27	/s	from Gioia et al., 2007 assuming the same value for fibronectin

For a spatiotemporal model, the center circular region of 49 compartments, which are shown in red in Figure 6A, was set as an invadopodium, into which all groups were embedded. Groups of “MMP2 inactivation” and “ECM degradation by MMP-2” were embedded into all compartments. Lateral diffusion of MT1-MMP was not employed, because our experimental data indicated that a

negligible amount of material was moved by lateral diffusion on the invadopodial membrane. TIMP-2, MMP-2 (both pro- and active-form) and their complex were assumed to diffuse within a 3D space with a diffusion coefficient of 10^{-15} m^2/s. This value is small in comparison to the coefficients for ordinary cytoplasmic soluble proteins (10^{-10}-10^{-12} m^2/s). However, the fibronectin, which was used as a component of ECM in our experiments, was not in a solution but was present as a gel resulting in much reduced diffusion. Therefore, we employed the small value of the diffusion coefficient.

References

Gioia M, Monaco S, Fasciglione GF, Coletti A, Modesti A, Marini S, Coletta M (2007) Characterization of the mechanisms by which gelatinase A, neutrophil collagenase, and membrane-type metalloproteinase MMP-14 recognize collagen I and enzymatically process the two alpha-chains. *J Mol Biol* **368:** 1101-1113

Joo J, Plimpton S, Martin S, Swiler L, Faulon JL (2007) Sensitivity analysis of a computational model of the IKK NF-kappaB IkappaBalpha A20 signal transduction network. *Ann N Y Acad Sci* **1115**: 221-239

Karagiannis ED, Popel AS (2004) A theoretical model of type I collagen proteolysis by matrix metalloproteinase (MMP) 2 and membrane type 1 MMP in the presence of tissue inhibitor of metalloproteinase 2. *J Biol Chem* **279:** 39105-39114

Murphy G, Willenbrock F (1995) Tissue inhibitors of matrix metalloendopeptidases. *Methods Enzymol* **248:** 496-510

Olson MW, Gervasi DC, Mobashery S, Fridman R (1997) Kinetic analysis of the binding of human matrix metalloproteinase-2 and -9 to tissue inhibitor of metalloproteinase (TIMP)-1 and TIMP-2. *J Biol Chem* **272:** 29975-29983

Toth M, Bernardo MM, Gervasi DC, Soloway PD, Wang Z, Bigg HF, Overall CM, DeClerck YA, Tschesche H, Cher ML, Brown S, Mobashery S, Fridman R (2000) Tissue inhibitor of metalloproteinase (TIMP)-2 acts synergistically with synthetic matrix metalloproteinase (MMP) inhibitors but not with TIMP-4 to enhance the (Membrane type 1)-MMP-dependent activation of pro-MMP-2. *J Biol Chem* **275:** 41415-41423
